# Healthcare Resource Utilization in Patients with Newly Diagnosed Atrial Fibrillation: A Global Analysis from the GARFIELD-AF Registry

**DOI:** 10.3390/healthcare11050638

**Published:** 2023-02-21

**Authors:** Lorenzo G. Mantovani, Paolo Cozzolino, Pietro Ferrara, Saverio Virdone, A. John Camm, Freek W. A. Verheugt, Jean-Pierre Bassand, Alexander G. G. Turpie, Werner Hacke, Gloria Kayani, Samuel Z. Goldhaber, Shinya Goto, Karen S. Pieper, Bernard J. Gersh, Keith A. A. Fox, Sylvia Haas, Martin van Eickels, Ajay K. Kakkar, on behalf of the GARFIELD-AF Investigators

**Affiliations:** 1 Center for Public Health Research, University of Milan-Bicocca, 20900 Monza, Italy; 2 Laboratory of Public Health, Istituto Auxologico Italiano—IRCCS, 20165 Milan, Italy; 3 Thrombosis Research Institute, London SW3 6LR, UK; 4 Cardiovascular Clinical Academic Group, St. George’s University of London, London SW17 0RE, UK; 5 Onze Lieve Vrouwe Gasthuis (OLVG), 1091 AC Amsterdam, The Netherlands; 6 Department of Cardiology, University of Besançon, 25030 Besançon, France; 7 Faculty of Health Sciences, McMaster University, Hamilton, ON L8S 4L8, Canada; 8 Department of Neurology, Ruprecht-Karls-University of Heidelberg, 69120 Heidelberg, Germany; 9 Division of Cardiovascular Medicine, Harvard Medical School, Boston, MA 02115, USA; 10 Department of Medicine (Cardiology), Tokai University School of Medicine, Isehara 259-1193, Japan; 11 Department of Cardiovascular Medicine, Mayo Clinic College of Medicine and Science, Rochester, NY 55905, USA; 12 Centre for Cardiovascular Science, University of Edinburgh, Edinburgh EH16 4TJ, UK; 13 Formerly Department of Medicine, Technical University of Munich, 81675 Munich, Germany; 14 Bayer AG, 13353 Berlin, Germany

**Keywords:** atrial fibrillation, healthcare resource utilization, inpatient care, outpatient care

## Abstract

The management of atrial fibrillation (AF), the most common sustained arrhythmia, impacts healthcare resource utilization (HCRU). This study aims to estimate global resource use in AF patients, using the GARFIELD-AF registry. A prospective cohort study was conducted to characterize HCRU in AF patients enrolled in sequential cohorts from 2012 to 2016 in 35 countries. Components of HCRU studied were hospital admissions, outpatient care visits, and diagnostic and interventional procedures occurring during follow-up. AF-related HCRU was reported as the percentage of patients demonstrating at least one event and was quantified as rate-per-patient-per-year (PPPY) over time. A total of 49,574 patients was analyzed, having an overall median follow-up of 719 days. Almost all patients (99.5%) had at least one outpatient care visit, while hospital admissions were the second most frequent medical contact, with similar proportions in North America (37.5%) and Europe (37.2%), and slightly higher in the other GARFIELD-AF countries (42.0%; namely Australia, Egypt, and South Africa). Asia and Latin America showed lower percentages of hospitalizations, outpatient care visits, and diagnostic and interventional procedures. Analyses of GARFIELD-AF highlighted the vast AF-related HCRU, underlying significant geographical differences in the type, quantity, and frequency of AF-related HCRU. These differences were likely attributable to health service availability and differing models of care.

## 1. Introduction

Atrial fibrillation (AF) is the most common arrhythmia and, with its progressively increasing prevalence, impacts public health and healthcare resource utilization (HCRU) [1,2]. AF affects approximately 37.5-million adults worldwide with about 400 new cases per 1-million inhabitants diagnosed annually [3]. AF patients are at increased risk for stroke and suffer an increase in morbidity and mortality [4]. AF’s association with hypercholesterolemia, diabetes mellitus, arterial hypertension, chronic kidney disease (CKD), dementia, obesity, and sleep apnea may confer a negative prognosis [4,5].

AF’s association with healthcare resource utilization (HCRU) presents a large economic burden [6]. AF is estimated to account for more than 1% of total healthcare expenditures in high-income countries, mostly attributable to hospitalization [7]. Other resource use and cost contributors include medical visits, emergency room (ER) admissions, and diagnostic and interventional procedures often required by AF patients (e.g., electrocardiography, laboratory tests, cardioversion, catheter ablation, etc.) [5,6,7,8].

Several studies have evaluated the multiple aspects of AF, including its HCRU burden, studied according to specific contexts, settings, or treatment options [9,10,11,12,13]. The objective of this study was to characterize the global HCRU in AF patients within the Global Anticoagulant Registry in the FIELD-AF (GARFIELD-AF). The GARFIELD-AF registry defines a non-interventional, observational study that characterized a global population of non-valvular AF patients. This multicenter global registry documented patients’ and sub-populations’ baseline characteristics, treatment strategies, and outcome measures by including five prospective cohorts of adult subjects who were newly diagnosed with non-valvular AF (diagnosed within the previous six weeks before enrolment) and having at least one additional risk factor for stroke. GARFIELD-AF also included a validation cohort of retrospective patients diagnosed with non-valvular AF between 6 and 24 months prior to enrolment [14,15].

## 2. Materials and Methods

### 2.1. Study Design and Data Source

A prospective cohort design was used to characterize resource utilization associated with the care of AF patients. The study investigated the GARFIELD–AF registry, an observational worldwide registry that prospectively and consecutively enrolled sequential cohorts of 52,167 newly diagnosed AF patients at risk of stroke from December 2009 to August 2016 in 35 countries. Eligible patients were aged 18 years or older, enrolled consecutively into five cohorts (representing seven years of enrollment, from 2010 to 2016) including ~10,000 participants each; the additional retrospective cohort (GARFIELD–AF Cohort 1) was excluded. Participants with a follow-up period of less than three months were excluded from the analysis. Data were extracted from the final study database lock (June 2019) in 2020. The GARFIELD–AF study design has been reported elsewhere [14,15]. Baseline patient characteristics—including demographic information, clinical conditions, risk stratification, and antithrombotic treatment—were collected at inclusion in the registry [14,15]. Risk stratification was documented through CHA_2_DS_2_-VASc (congestive heart failure, hypertension, age ≥ 75 years [doubled], diabetes, stroke [doubled], vascular disease, age 65–74 years, and sex category [female]). Follow-up data on treatments and outcomes were collected at four monthly intervals up to 24 months. GARFIELD–AF data were captured using an electronic case report form (eCRF) designed by Dendrite Clinical Systems Ltd. (Henley-on-Thames, UK). Oversight of operations and data management were performed by the coordinating center (Thrombosis Research Institute, London, UK). The study is registered at ClinicalTrials.gov (unique identifier: NCT01090362). Patients were selected from multiple healthcare settings and were registered by the identifying clinician registered using the eCRF. Data were collected from five clinical sources associated with the patient (i.e., hospital, emergency department, anticoagulation clinic, stroke unit, and office-based settings such as general or family practitioners, cardiologists, and internists) through a review of patient notes and clinical records [14]. Data on HCRU and changes in medication treatment were stored in a dedicated follow-up and events dataset.

### 2.2. Outcomes Measures and Definitions

HCRU in AF patients was evaluated focusing on medical contacts and is reported as the proportion and frequency of at least one event (besides the recruitment visit, which was excluded from the analysis). Events included in the analysis of HCRU studied were those linked to AF and its sequalae and collected during follow-up visits as per study protocol and according to standardized outcome definitions [14]. Studied HCRU items include hospital admissions, outpatient hospital attendance, ER admissions, family doctor visits, stroke unit admissions and office-based specialist visits, and diagnostic and interventional procedures occurring during the follow-up period. General practitioner visits, office-based specialist visits, and hospital-based outpatient visits were grouped as “outpatient care visits,” to adequately compare information from different countries and settings. Diagnostic and interventional procedures covered all those derived from follow-up events, including those specific to AF (such as electrical cardioversion and ablation), methods for pulmonary embolism diagnosis (e.g., computed tomography scan, magnetic resonance imaging scan, and invasive angiography) and interventions required for cardiovascular diseases (including percutaneous coronary intervention [PCI] bare metal stent, PCI drug eluting stent, PCI balloon angioplasty, coronary artery bypass graft, valve replacement, pacemaker, and carotid stent). Data on medication use were not included in this study, as this has been evaluated in previous analyses of the GARFIELD-AF registry [16,17,18].

For the purpose of this analysis, patients were divided into two groups according to the enrolment cohorts, which allowed to account for possible differences in HCRU over the whole study period. Group A included participants recruited into GARFIELD–AF Cohorts 2 and 3 from 2010 to 2013; Group B included those in Cohorts 4 to 6 from 2013 to 2016. In particular, we split patients into two 3-year timeslots since, by Cohort 3, the non-Vitamin K antagonist oral anticoagulants (NOAC) were approved in most of the countries included in the GARFIELD-AF registry. In addition, there has been an increased use in newly diagnosed patients with AF receiving guideline-recommended treatment [18].

The 35 countries within the registry were grouped by geographical region, according to the classification provided by the GARFIELD–AF dataset used: Asia (China, India, Japan, Korea, Singapore, Thailand, Turkey, and United Arab Emirates), Europe (Austria, Belgium, Czech Republic, Denmark, Finland, France, Germany, Hungary, Italy, The Netherlands, Norway, Poland, Russia, Spain, Sweden, Switzerland, Ukraine, and United Kingdom), Latin America (Argentina, Brazil, Chile, and Mexico), North America (Canada and United States), and other GARFIELD–AF countries (Australia, Egypt, and South Africa, henceforth defined as “others”).

### 2.3. Statistical Analysis

Continuous variables were described with mean and median as central tendency measures, while standard deviation (SD) and interquartile range (IQR) were described as dispersion measures. Categorical variables have been presented using frequency and percentage. The Student’s t test was used to assess differences between continuous variables, and the Chi square (*χ*^2^) or Fisher’s exact tests were used when needed to assess differences between categories.

AF-related HCRU was reported as the percentage of patients having at least one event included in the analyses and quantified as rate-per-patient-per-year (PPPY). Region-specific HCRU rates were subsequently compared using a Poisson regression model, which was adjusted for possible known confounders and modifiers collected in the registry, such as sex, age at enrolment, type of AF (i.e., (i) paroxysmal: AF that lasts less than 7 days and resolves spontaneously or with intervention; (ii) persistent: AF episode that continues for more than 7 days, irrespective of whether the episode was terminated by cardioversion or if it self-terminated; (iii) permanent: when AF is accepted by the patient (and physician) and a rate-control strategy is needed; or (iv) new onset—unclassified), AF therapy (i.e., antiplatelet [AP], alone or in combination with Vitamin K antagonists [VKA] or NOAC), comorbidities, prior transient ischemic attack, prior bleeding, CHA_2_DS_2_-VASc score, country income level (i.e., high, upper-middle, or lower-middle), and healthcare system payer (i.e., single payer, universal public insurance, public-private insurance, or private insurance). Results are expressed as incidence rate ratios (IRR) with 95% confidence intervals (95% CI). All *p*-values were two-sided, with values <0.05 considered statistically significant. Analyses were performed using STATA statistical software version 13.1 [19].

## 3. Results

### 3.1. Baseline Sample Characteristics

This study involved a total of 49,574 patients, with an overall median follow-up period of 719 days (IQR, 597–730). The cohort was mainly constituted of subjects enrolled in Europe (56.5%) and Asia (28.4%), with a median age of 71 years (IQR, 63–78). More than half of the participants were men (55.7%). Prior bleeding was reported in 2.5%; transient ischemic attack in 4.4%; diabetes in 22.4%. A complete overview of patient demographics and clinical characteristics, according to region, is listed in Table 1.

Differences according to the cohorts of enrollment are presented in Table 2. The two cohort groups differed in almost all the baseline clinical characteristics.

### 3.2. Healthcare Resource Utilization

The vast majority of patients (99.5%) had at least one outpatient care visit, excluding the enrollment medical contact. Hospitalization was the second-most frequent medical contact, with almost one-third (30.4%) of patients having at least one hospital visit. Higher proportions of patients with more than one hospitalization were observed in North America (37.5%), Europe (37.2%), and others (42.0%). Of these, stroke unit admissions accounted for around 1% in all groups. Higher numbers of procedures and ER admissions were registered in North America with, respectively, 25.1% and 31.0% patients. A lower proportion of patients with at least one ER admission was seen in Asia (8.1%), and a lower proportion of ER procedures was recorded in Latin America (7.5%). Table 3 reports the number of GARFIELD–AF participants with at least one HCRU event.

**Figure 1:**
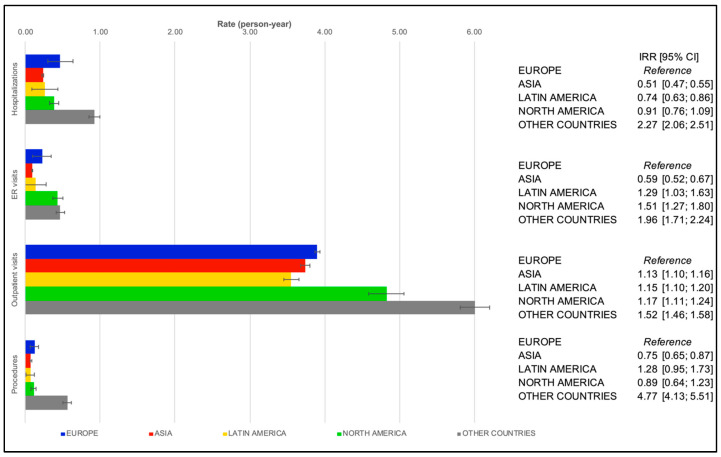
Regression-adjusted per-patient-per-year rates of healthcare resource utilization in patients with atrial fibrillation in cohort Group A (GARFIELD–AF Cohorts 1 and 2) by region. Abbreviations: IRR, incidence rate ratio; 95% CI, 95% confidence interval; ER, emergency room.

**Figure 2:**
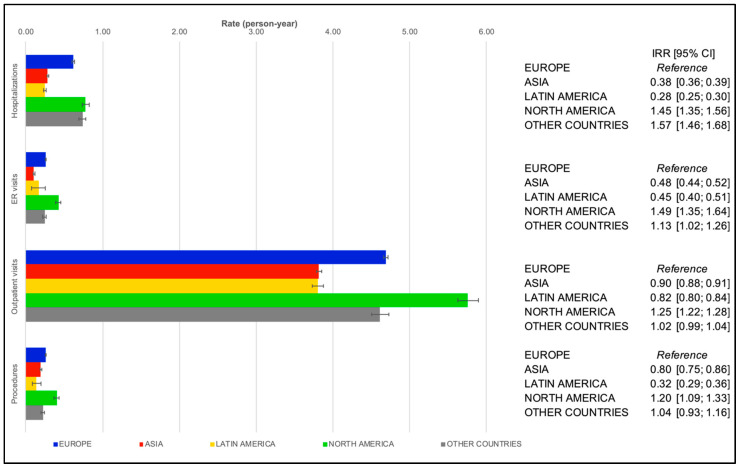
Regression-adjusted per-patient-per-year rates of healthcare resource utilization in patients with atrial fibrillation in cohort Group B (GARFIELD–AF Cohorts 4 to 6) by region. Abbreviations: IRR, incidence rate ratio; 95% CI, 95% confidence interval; ER, emergency room.

The cohort groups’ PPPYs results aggregated by region are presented in Figure 1 and Figure 2. Outpatient care visits were the most frequent event in both groups (i.e., participants enrolled between 2010 and 2013 and those enrolled from 2013 to 2016). Large variations in the type of other medical contacts were observed across regions and between the two cohort groups.

In Group A (GARFIELD–AF Cohorts 2 and 3), patients with higher PPPY rates for procedures and hospitalizations were in Europe and others, while lesser values for both events were seen in Asia. In the latter, the lowest PPPY rate for ER visits was also registered. Patients in Europe showed lower PPPY rates for outpatient visits (Figure 1).

Overall, AF-related HCRU trends in Group B (GARFIELD–AF Cohorts 4 to 6) mirrored those in Group A, with narrower regional differences as compared with the previous (Figure 2). Compared with Europe, patients in North America showed higher HRCU rates for all medical contacts, and those in Latin America and Asia showed lower ones.

## 4. Discussion

In this real-world observational study, we combined global data from the GARFIELD–AF registry on the estimated HCRU in AF patients and compared it among regions and over time. Our findings highlighted the extensive resources utilized in almost 50,000 subjects from 35 countries worldwide. Important disparities still exist in their utilization among patients in the five regions after controlling for various confounders, such as patients’ characteristics and clinical status, as well as societal aspects (for instance, country income level and healthcare system payer).

All HCRU components showed an overlapping pattern across the five regions in the two study groups, but frequencies changed across cohorts. In particular, the analyses highlighted narrower regional differences in the second period along with the differences shown between the two groups as compared with the first one. Overall, these changes may indicate an increasingly progressive concordance with evidence-based guidelines for patients newly diagnosed with AF across countries, mirroring trends seen in previous GARFIELD–AF research. It appears that clinical practice and treatment of AF patients has become more uniform over time, likely due to a wider use of NOACs and specific AF procedures, such as electrical cardioversion and ablation [20,21].

As regards to the regional distribution of AF-related HCRU, a primary reason for these differences may be the availability of services and the differing models of healthcare and AF-care organization, beyond differences in healthcare system and payer [22]. In certain settings, gate-keeping systems—such as an initial visit to a general practitioner for access to specialist care or the presence of transitional care facilities—influenced the patient’s use of services. A previous analysis conducted in Latin American countries included in the GARFIELD–AF registry has suggested inadequate management of AF patients, with therapy underuse attributable to physician choice, difficulties in accessing healthcare, adverse economic conditions, and lower educational levels [23]. Additionally, access to primary and cardiology care in rural communities may be a recurring challenge for older and disabled AF adults, resulting in gaps in access to health services [24].

Another factor influencing HCRU variations across regions is inconsistent patient demographics, particularly the population age structure. These differing age structures may persist also after appropriate confounder correction [25]. Thus, greater numbers and frequencies of medical contacts may be at least partly attributed to the larger proportion of elderly people in some countries. Similarly, AF epidemiological metrics should be considered in the interpretation of our results. Although global rates are relatively stable, higher and more premature mortality due to AF was shown in low- and middle-income countries [4,23,26]. In contrast, a lower risk of death in Asia and Europe compared with other regions is a common observation, likely linked to the highly protective healthcare system and easier access to services in these regions [27]. Living in North America or Latin America was instead associated with a higher risk of early death [27]. A bias toward lower reported medical contacts may exist in countries where such services lack or are underused, resulting in a suboptimal level of care.

When analyzing the type of healthcare contacts, it is worth noting that hospitalizations account for higher HCRU rates. Drivers for urgent and elective hospitalization in AF patients have been extensively described in the literature, and include cardiovascular and non-traditional risk factors, as well as considerable rates of readmission, particularly in comorbid, higher CHA_2_DS_2_VASc score, and post-ablated AF patients [28,29,30]. Overall, inpatient care is the main determinant of healthcare costs associated with AF. Thus, further research is needed to develop specific effective transitional and integrated care interventions [6,7,29].

In summary, although marked differences in resource use for AF patient care were observed worldwide, using the expansive GARFIELD–AF registry, our findings suggest that AF substantially contributes to resource consumption with a subsequent important impact on healthcare expenditure worldwide [2,29,31].

The management of AF is complex, and convergence towards guideline-directed care is crucial to maximize patient’s benefit from tailored treatment options. Yet, implanting integrated AF care models has been proven to reduce disease and resource burden of AF [29]. In this sense, our findings may serve as actionable indicators of novel value-based organizational approaches to support changes in the management of AF.

This paper has a number of strengths and weaknesses. The design features of GARFIELD–AF registry include the random selection of sites and the enrolment of patients without exclusion according to comorbidities or treatment that ensures, respectively, the representativeness of the national care settings and population aimed to study, thus providing reliable estimates of research outcomes. Despite these strengths, this research should be interpreted in the context of its limitations. The study did not consider other possible unmeasured confounders, which may influence HCRU in AF patients. However, we included those mainly associated with the outcomes, and the use of robust statistical analysis allowed us to balance factors potentially correlated to such confounders. The reported burden of resource consuming was quantified excluding medication use, which was previously characterized in other GARFIELD–AF studies [16,17,18]. The differences in healthcare systems and organization across the countries included in the GARFIELD–AF registry may reflect variability in types, amounts, and patterns of HCRU events.

## 5. Conclusions

Within the GARFIELD–AF registry, a vast amount of HCRU was documented in AF patients from 35 countries worldwide. Important geographical differences exist in the type, quantity, and frequency of HCRU in patients with AF. Changes in AF care and variable adherence to evidence-based guidelines determined different patterns of HCRU, with a trend toward convergence of clinical practices over time.

## Figures and Tables

**Table 1:** Patient demographics and clinical characteristics.

	Total	Asia	Europe	Latin America	North America	Other GARFIELD–AF Countries
N (%)	49,574 (100.00)	14,059 (28.36)	27,987 (56.45)	4004 (8.08)	1554 (3.13)	1970 (3.97)
**Cohort**						
Group A (C2-3)	16,459 (33.20)	3711 (26.40)	10,449 (37.34)	1390 (34.72)	331 (21.30)	578 (29.34)
Group B (C4-6)	33,115 (66.80)	10,348 (73.60)	17,538 (62.66)	2614 (65.28)	1223 (78.70)	1392 (70.66)
**Sex**						
Male	27,586 (55.65)	8251 (58.69)	15,293 (54.64)	2106 (52.60)	849 (54.63)	1087 (55.18)
Female	21,988 (44.35)	5808 (41.31)	12,694 (45.36)	1898 (47.40)	705 (45.37)	883 (44.82)
**Age**						
Mean (SD)	69.62 (11.48)	67.54 (12.00)	70.73 (10.90)	69.77 (11.92)	71.05 (11.84)	67.32 (12.04)
Median (IQR)	71 (63–78)	69 (60–76)	72 (64–79)	71 (63–78.5)	72 (64–80)	68 (60–76)
**Comorbid conditions**						
Diabetes						
No	38,458 (77.58)	10,903 (77.55)	21,975 (78.52)	3011 (75.20)	1143 (73.55)	1426 (72.39)
Yes	11,116 (22.42)	3156 (22.45)	6012 (21.48)	993 (24.80)	411 (26.45)	544 (27.61)
Chronic kidney disease						
No	22,753 (45.90)	7941 (56.48)	11,055 (39.50)	1876 (46.85)	926 (59.59)	955 (48.48)
I	9994 (20.16)	2702 (19.22)	6034 (21.56)	809 (20.20)	164 (10.55)	285 (14.47)
II	5896 (11.89)	1020 (7.26)	4314 (15.41)	192 (4.80)	112 (7.21)	258 (13.10)
III/IV	4918 (9.92)	960 (6.83)	3387 (12.10)	241 (6.02)	125 (8.04)	205 (10.41)
V	226 (0.46)	108 (0.77)	74 (0.26)	26 (0.65)	11 (0.71)	7 (0.36)
Unknown	5786 (11.67)	1328 (9.45)	3123 (11.16)	859 (21.45)	216 (13.90)	260 (13.20)
Missing	1 (0.00)	0 (0.00)	0 (0.00)	1 (0.02)	0 (0.00)	0 (0.00)
Hypercholesterolemia						
No	27,841 (56.16)	9751 (69.36)	14,317 (51.16)	2327 (58.12)	623 (40.09)	823 (41.78)
Yes	20,257 (40.86)	4020 (28.59)	12,778 (45.66)	1468 (36.66)	904 (58.17)	1087 (55.18)
Unknown	1476 (2.98)	288 (2.05)	892 (3.19)	209 (5.22)	27 (1.74)	60 (3.05)
Cirrhosis						
No	48,647 (98.13)	13,784 (98.04)	27,541 (98.41)	3912 (97.7)	1505 (96.85)	1905 (96.70)
Yes	272 (0.55)	92 (0.65)	134 (0.48)	14 (0.35)	14 (0.90)	18 (0.91)
Unknown	655 (1.32)	183 (1.30)	312 (1.11)	78 (1.95)	35 (2.25)	47 (2.39)
Congestive Heart Failure						
No	39,688 (80.06)	11,274 (80.19)	22,237 (79.45)	3200 (79.92)	1323 (85.14)	1654 (83.96)
Yes	9886 (19.94)	2785 (19.81)	5750 (20.55)	804 (20.08)	231 (14.86)	316 (16.04)
Vascular Disease						
No	42,209 (85.14)	12,400 (88.20)	23,553 (84.16)	3425 (85.54)	1285 (82.69)	1546 (78.48)
Yes	7365 (14.86)	1659 (11.80)	4434 (15.84)	579 (14.46)	269 (17.31)	424 (21.52)
**Type of atrial fibrillation**						
Permanent	6285 (12.68)	1195 (8.50)	4193 (14.98)	643 (16.06)	35 (2.25)	219 (11.12)
Persistent	7427 (14.98)	2438 (17.34)	4104 (14.66)	597 (14.91)	97 (6.24)	191 (9.70)
Paroxysmal	13,739 (27.71)	5169 (36.77)	6933 (24.77)	1050 (26.22)	332 (21.36)	255 (12.94)
New onset (unclassified)	22,123 (44.63)	5257 (37.39)	12,757 (45.58)	1714 (42.81)	1090 (70.14)	1305 (66.24)
**Stroke prophylaxis**						
AP or none	15,933 (32.14)	6013 (42.77)	7431 (26.55)	1484 (37.06)	500 (32.18)	505 (25.63)
VKA ± AP	19,352 (39.04)	4140 (29.45)	12,396 (44.29)	1580 (39.46)	341 (21.94)	895 (45.43)
NOACs ± AP	13,598 (27.43)	3752 (26.69)	7733 (27.63)	874 (21.83)	694 (44.66)	545 (27.66)
Unknown	691 (1.39)	154 (1.10)	427 (1.53)	66 (1.65)	19 (1.22)	25 (1.27)
**History of bleeding**						
No	48,157 (97.14)	13,723 (97.61)	27,230 (97.3)	3841 (95.93)	1470 (94.59)	1893 (96.09)
Yes	1237 (2.50)	230 (1.64)	712 (2.54)	158 (3.95)	72 (4.63)	65 (3.30)
Unknown	180 (0.36)	106 (0.75)	45 (0.16)	5 (0.12)	12 (0.77)	12 (0.61)
**Prior transient ischemic attack**						
No	47,138 (95.09)	13,631 (96.96)	26,460 (94.54)	3815 (95.28)	1457 (93.76)	1775 (90.10)
Yes	2183 (4.40)	302 (2.15)	1451 (5.18)	173 (4.32)	81 (5.21)	176 (8.93)
Unknown	253 (0.51)	126 (0.90)	76 (0.27)	16 (0.40)	16 (1.03)	19 (0.96)
**CHA_2_DS_2_-VASc score**						
0	1366 (2.76)	684 (4.87)	470 (1.68)	115 (2.87)	46 (2.96)	51 (2.59)
1	6072 (12.25)	2300 (16.36)	2904 (10.38)	442 (11.04)	175 (11.26)	251 (12.74)
2	9950 (20.07)	3116 (22.16)	5428 (19.39)	728 (18.18)	286 (18.40)	392 (19.90)
3	11,954 (24.11)	3181 (22.63)	7010 (25.05)	933 (23.30)	366 (23.55)	464 (23.55)
4	10,837 (21.86)	2584 (18.38)	6481 (23.16)	982 (24.53)	366 (23.55)	424 (21.52)
5	5624 (11.34)	1356 (9.65)	3399 (12.14)	470 (11.74)	188 (12.10)	211 (10.71)
6–9	3771 (7.61)	838 (5.96)	2295 (8.20)	334 (8.34)	127 (8.17)	177 (8.98)

Abbreviations: C, enrolment cohort; SD, standard deviation; IQR, interquartile range; AP, antiplatelet therapy; VKA, vitamin K antagonists; NOAC, non-vitamin K antagonist oral anticoagulants.

**Table 2:** Differences in patient characteristics according to cohort groups.

	Cohort Group A	Cohort Group B	Total	*p*-Value
N (%)	16,459 (33.20)	33,115 (66.80)	49,574 (100.0)	
**Sex**				0.316
Male	9211 (55.96)	18,375 (55.49)	27,586 (55.65)	
Female	7248 (44.04)	14,740 (44.51)	21,988 (44.35)	
**Age**				0.139
Mean (SD)	69.73 (11.40)	69.57 (11.52)	69.62 (11.48)	
Median (IQR)	71 (63-78)	71 (62–78)	71 (63–78)	
**Comorbid conditions**				
Diabetes				0.247
No	12,819 (77.88)	25,639 (77.42)	38,458 (77.58)	
Yes	3640 (22.12)	7476 (22.58)	11,116 (22.42)	
Chronic kidney disease *				<0.001
None	93 (0.57)	22,660 (68.43)	22,753 (45.90)	
I	8099 (49.21)	1895 (5.72)	9994 (20.16)	
II	2434 (14.79)	3462 (10.45)	5896 (11.89)	
III/IV	1629 (9.90)	3289 (9.93)	4918 (9.92)	
V	70 (0.43)	156 (0.47)	226 (0.46)	
Unknown	4134 (25.12)	1652 (4.99)	5786 (11.67)	
Missing	0 (0.00)	1 (0.00)	1 (0.00)	
Hypercholesterolemia				<0.001
No	9765 (59.33)	18,076 (54.59)	27,841 (56.16)	
Yes	6689 (40.64)	13,568 (40.97)	20,257 (40.86)	
Unknown	5 (0.03)	1471 (4.44)	1476 (2.98)	
Cirrhosis				<0.001
No	16,365 (99.43)	32,282 (97.48)	48,647 (98.13)	
Yes	83 (0.50)	189 (0.57)	272 (0.55)	
Unknown	11 (0.07)	644 (1.94)	655 (1.32)	
Congestive Heart Failure				0.001
No	13,041 (79.23)	26,647 (80.47)	39,688 (80.06)	
Yes	3418 (20.77)	6468 (19.53)	9886 (19.94)	
Vascular Disease				0.345
No	14,049 (85.36)	28,160 (85.04)	42,209 (85.14)	
Yes	2410 (14.64)	4955 (14.96)	7365 (14.86)	
**Type of atrial fibrillation**				<0.001
Permanent	2160 (13.12)	4125 (12.46)	6285 (12.68)	
Persistent	2556 (15.53)	4871 (14.71)	7427 (14.98)	
Paroxysmal	4199 (25.51)	9540 (28.81)	13,739 (27.71)	
New onset (unclassified)	7544 (45.84)	14,579 (44.03)	22,123 (44.63)	
**Stroke prophylaxis**				<0.001
VKA ± AP	8161 (49.58)	11,191 (33.79)	19,352 (39.04)	
NOACs ± AP	1781 (10.82)	11,817 (35.68)	13,598 (27.43)	
None ± AP	6232 (37.86)	9701 (29.29)	15,933 (32.14)	
Unknown	285 (1.73)	406 (1.23)	691 (1.39)	
**History of bleeding**				<0.001
No	15,979 (97.08)	32,178 (97.17)	48,157 (97.14)	
Yes	472 (2.87)	765 (2.31)	1237 (2.50)	
Unknown	8 (0.05)	172 (0.52)	180 (0.36)	
**Prior transient ischemic attack**				<0.001
No	15,582 (94.67)	31,556 (95.29)	47,138 (95.09)	
Yes	870 (5.29)	1313 (3.96)	2183 (4.40)	
Unknown	7 (0.04)	246 (0.74)	253 (0.51)	
**CHA_2_DS_2_-VASc score**				<0.001
0	386 (2.35)	980 (2.96)	1366 (2.76)	
1	1951 (11.85)	4121 (12.44)	6072 (12.25)	
2	3218 (19.55)	6732 (20.33)	9950 (20.07)	
3	3975 (24.15)	7979 (24.09)	11,954 (24.11)	
4	3610 (21.93)	7227 (21.82)	10,837 (21.86)	
5	1947 (11.83)	3677 (11.10)	5624 (11.34)	
6–9	1372 (8.34)	2399 (7.24)	3771 (7.61)	

* Major discrepancies in rates of chronic kidney disease between groups may be attributable to changes in disease definition that occurred after the enrollment of GARFIELD–AF Cohort 2. Abbreviations: SD, standard deviation; IQR, interquartile range; AP, antiplatelet therapy; VKA, Vitamin K antagonists; NOAC, non-Vitamin K antagonist oral anticoagulants.

**Table 3:** Patients with at least one medical contact.

HCRU Events * (N [%])	Total	Asia	Europe	Latin America	North America	Other GARFIELD–AF Countries
Hospitalizations	15,046 (30.35)	2617 (18.61)	10,412 (37.20)	607 (15.16)	582 (37.45)	828 (42.03)
ER admissions	8129 (16.40)	1137 (8.09)	5637 (20.14)	434 (10.84)	482 (31.02)	439 (22.28)
Procedures ^	7167 (14.46)	1540 (10.95)	4527 (16.18)	302 (7.54)	390 (25.10)	408 (20.71)

* Outpatient care visits were not reported since all patients had at least one. ^ Type and number of interventional procedures included in the analysis are detailed in Table S1 (supplementary materials). Abbreviations: HCRU, healthcare resource utilization; ER, emergency room.

## Data Availability

Data are available on reasonable request. The data underlying this article will be shared on reasonable request from Karen S. Pieper (kpieper@tri-london.ac.uk).

## Supplementary Materials

The following are available online at https://www.mdpi.com/article/10.3390/healthcare11050638/s1: Section S1, Complete list of GARFIELD-AF investigators. Section S2, Appendix to Results: Table S1: Interventional procedures included in the analysis.
