# Supplementary material for: Healthcare Resource Utilization in Patients with Newly Diagnosed Atrial Fibrillation: A Global Analysis from the GARFIELD-AF Registry
Source: Healthcare (Basel). 2023 Feb 21;11(5):638. doi: 10.3390/healthcare11050638 (PMC10000823; doi:10.3390/healthcare11050638)
Supplement: Supplementary file 1 [file healthcare-11-00638-s001.zip › healthcare-2209622-supplementary.pdf]

Supplementary Materials

Healthcare resource utilization in patients with newly diagnosed atrial fibrillation: A global analysis from the GARFIELD-AF registry

Lorenzo G. Mantovani [1,2,*], Paolo Cozzolino [1,†], Pietro Ferrara [2,†], Saverio Virdone [3], A. John Camm [4], Freek W.A. Verheugt [5], Jean-Pierre Bassand [3,6], Alexander G.G. Turpie [7], Werner Hacke [8], Gloria Kayani [3], Samuel Z. Goldhaber [9], Shinya Goto [10], Karen S. Pieper [3], Bernard J. Gersh [11], Keith A.A. Fox [12], Sylvia Haas [13], Martin van Eickels [14], Ajay Kakkar [3] for the GARFIELD-AF investigators

1 Center for Public Health Research, University of Milan-Bicocca, 20900 Monza, Italy
2 Istituto Auxologico Italiano—IRCCS, 20165 Milan, Italy
3 Thrombosis Research Institute, SW3 6LR London, United Kingdom
4 Cardiovascular Clinical Academic Group, St. George's University of London, SW17 0RE London, United Kingdom
5 Onze Lieve Vrouwe Gasthuis (OLVG), 1091 AC Amsterdam, The Netherlands
6 University of Franche-Comté, 25030 Besançon, France
7 McMaster University, L8S 4L8 Hamilton, Canada
8 Department of Neurology, Ruprecht-Karls-University of Heidelberg, 69120 Heidelberg, Germany
9 Brigham and Women's Hospital and Harvard Medical School, 02115 Boston, USA
10 Tokai University, 259-1207 Kanagawa, Japan
11 Department of Cardiovascular Medicine, Mayo Clinic College of Medicine and Science, 55905 Rochester, USA
12 Centre for Cardiovascular Science, University of Edinburgh, EH16 4TJ Edinburgh, United Kingdom
13 Formerly Department of Medicine, Technical University of Munich, 81675 Munich, Germany
14 Bayer AG, 13353 Berlin, Germany
* Correspondence: lorenzo.mantovani@unimib.it (L.G.M.)

Supplementary materials

S1. Complete list of GARFIELD-AF Principle Investigators

S1.1. Global Steering Committee

Ajay K. Kakkar (UK) (Chair), Jean-Pierre Bassand (France), A. John Camm (UK), David A. Fitzmaurice (UK), Samuel Z. Goldhaber (USA), Shinya Goto (Japan), Sylvia Haas (Germany), Werner Hacke (Germany), Lorenzo G. Mantovani (Italy), Frank Misselwitz (Germany), Karen S. Pieper (USA), Alexander G.G. Turpie (Canada), Martin van Eickels (Germany), Freek W.A. Verheugt (the Netherlands).

S1.2. Publications Committee

A. John Camm (UK) (Chair), Jean-Pierre Bassand (France), Samuel Z. Goldhaber (USA), Sylvia Haas (Germany), Gloria Kayani (UK), Lorenzo G. Mantovani (Italy).

S1.3. Audit Committee

Keith A.A. Fox (UK), Bernard J. Gersh (USA).

S1.4. GARFIELD-AF National Coordinators

Hector Lucas Luciardi (Argentina), Harry Gibbs (Australia), Marianne Brodmann (Austria), Frank Cools (Belgium), Antonio Carlos Pereira Barretto (Brazil), Stuart J. Connolly, Alex Spyropoulos, John Eikelboom (Canada), Ramon Corbalan (Chile), Dayi Hu (China), Petr Jansky (Czech Republic), Jørn Dalsgaard Nielsen (Denmark), Hany Ragy

(Egypt), Pekka Raatikainen (Finland), Jean-Yves Le Heuzey (France), Harald Darius (Germany), Matyas Keltai (Hungary), Sanjay Kakkar and Jitendra Pal Singh Sawhney (India), Giancarlo Agnelli and Giuseppe Ambrosio (Italy), Yukihiro Koretsune (Japan), Carlos Jerjes Sánchez Díaz (Mexico), Hugo Ten Cate (the Netherlands), Dan Atar (Norway), Janina Stepinska (Poland), Elizaveta Panchenko (Russia), Toon Wei Lim (Singapore), Barry Jacobson (South Africa), Seil Oh (South Korea), Xavier Viñolas (Spain), Marten Rosenqvist (Sweden), Jan Steffel (Switzerland), Pantep Angchaisuksiri (Thailand), Ali Oto (Turkey), Alex Parkhomenko (Ukraine), Wael Al Mahmeed (United Arab Emirates), David Fitzmaurice (UK), Samuel Z. Goldhaber (USA).

S1.5. GARFIELD-AF National Investigators:

China

Dayi Hu, Yihong Sun, Lei Li, Wenling Liu, Yuanfeng Gao, Kangning Chen, Hong Zhai, Yusheng Zhao, Ran Zhang, Huaiqin Zhang, Xiao Chen, Jiyan Chen, Tingting Liu, Kan Wang, Xiaosheng Hu, Shiping Cao, Qiong Zhan, Jingshan Yang, Xingfu Huang, Daowen Wang¸ Ting Yu, Xiaoxue Yan, Yang Bai, Yuejin Yang, Xuesi Wu Qian Zhang, Zhicheng Jing, Xiaoliang Luo, Zhicheng Jing, Xiaojin Gao, Weihua Li, Wuyang Zheng, Hui Li, Yanhong Li, Hongying Yu, Tiebing Song, Shanshan Feng, Yuehui Yin, Xianbin Lan, Bei Zhou; Guizhou Tao, Ge Tian, Xiaojing Shi, Ping Yang, Bing Li, Yingmin Chen, Shenghu He, Yi Zhang, Yong Wang, Jing Li, Guosheng Fu, Yang Ye, Xia Sheng, Xin Li, Tongguo Wu, Jin Chen, Cancan Zuo, Xiaobi Guo, Xiaoshu Cheng, Qinmei Xiong, Aijuan Xu, Juxiang Li, Jianhua Yu; Xiaowei Yan, Ruiyi Xu, Xue Lin; Ruiping Zhao, Yangyang Liu, Rina Wu, Moshui Chen, Tianyi Ma, Yixue Zhang, Zhihong Zhou, Xiaoai He, Longgen Xiong, Li Wang, Chuanfang Cheng, Chengfeng Luo, Zhicheng Lu, Ping Chen, Ying Wang, Yang Jiao, Xinyi Zhu, Ying Guo, Qinghua Fu, Qin Yang, Na Li, Jianqiang Peng, Qiong Xie, Li Xue, Weina Guo, Ruhua He, Lin Chen, Guoshan Zhang, Zhiming Yang, Fengzhi Wang, Shuai Feng, Lin Yang, Jinxiu Zhang, Chunlin Bai.

India

Praveen Jadhavm, Priyanka Dhakrao, Deepak Ghumare, Raghava Sarma, Naga Malleswara Rao, Govind Kulkarni, Unnati Joshi, Bhakti Deshpande, Shankar G Panse, Anjali Sable, Amol Lawande, Prakash Chandwani, Mahendra Sharma, Rasesh Atulbhai Pothiwala, Dhrumi Shah, Nehal Sadhu, Pratik Dwivedi, Mohanan Padinhare Purayil, Divin Davies, Manoj Earath, Kamaldeep Chawla, Amish Ganatra, Khyati Patel, Veerappa Annasaheb Kothiwale, James Kot, Kinjal Shah,Vineeta Dhyani, Bagirath Raghuraman, Sudheer Garapati, Ayyappa N, Subramani Krishnappa, Vinod Madan Vijan, Mahesh Dargude, Jitendra Sawhney, Somya Duhan, Ganapathi Bantwal,Sudha Suresh, Aziz Khan, Puja Sherke, Satish Giradkar, Kajal Gaikwad, Reshma Kohade, Alka Gedam, Ramdhan Meena, Jugal Gupta, Vipin Jain, Shweta Paliwal, Manojkumar Chopada, Bhushan Suryawanshi, Priyanka More, Anand Rokade, Sunitha Abraham, Rajendran Karthikeyan, Anusha Kotha, Vikas Bisne, Bhowari Jamunkar, Neha Madarkar, Darshana Nandekar, Ruchieta Kishty, Govindan Vijayaraghavan, Kavitha VC, Debabrata Roy, Partha Bhattacharjee, Anjuli Barai, Rajashekhar Durgaprasad, Latheef Kasala, Vanaja Vanajakshamma, A.G. Ravi Shankar, Rahul ER, Kanuri Prashanth, Prasad Shiva, Dilip Teja Naidu, Sunil Kumar, Dilipa Naik, Manasa Rao, Dinesh Jain, Sukhjeet Kaur, Anju Jain, Aarushi Jain, Kartikeya Bhargava, Dhriti Adak, Krishan Sharma, Kuldeep Chauhan, Vinay Kumar, Devina Igoor, Manjappa M, Vinushree K, Rathna RL, Haseena Begum, Vaibhavi PS, Bharath Kumar, Udigala Madappa Nagamalesh, Dayakar P, Sanjana Xavier, Swathi Balaraju, Vijay Kumar, Beena Lokesh, Rajeeve Kumar Rajput, Swati Sharma, Huma Yusuf, Arti Malik, Swati Singhal.

Japan

Yukihiro Koretsune, Seishu Kanamori, Kenichi Yamamoto, Koichiro Kumagai, Yosuke Katsuda, Keiki Yoshida, Kemji Sadamatsu, Fumitoshi Toyota, Yuji Mizuno, Ikuo Misumi, Hiroo Noguchi, Shinichi Ando, Tetsuro Suetsugu, Masahiro Minamoto, Hiroyuki Oda, Susumu Adachi, Kei Chiba, Hiroaki Norita, Makoto Tsuruta, Takeshi Koyanagi, Kunihiko Yamamoto, Hiroshi Ando, Takayuki Higashi, Megumi Okada, Shiro Azakami, Shinichiro Komaki, Kenshi Kumeda, Takashi Murayama, Jun Matsumura, Yurika Oba, Ryuji Sonoda, Kazuo Goto, Kotaro Minoda, Yoshikuni Haraguchi, Hisakazu Suefuji, Hiroo Miyagi, Hitoshi Kato, Tsugihiro Nakamura, Tadashi Nakamura, Hidekazu Nandate, Ryuji Zaitsu, Yoshihisa Fujiura, Akira Yoshimura, Hiroyuki Numata, Jun Ogawa, Hiroshi Tatematsu, Yasuyuki Kamogawa, Kinshiro Murakami, Yutaka Wakasa, Masanori Yamasawa, Hiromitsu Maekawa, Sumihisa Abe, Hajime Kihara, Satoru Tsunoda, Katsumi Saito, Hiroki Tachibana, Ichiro Oba, Takashi Kuwahata, Satoshi Higa, Masamichi Gushiken, Takuma Eto, Hidetoshi Chibana, Kazuaki Fujisawa, Yuhei Shiga, Hirokuni Sumi, Toshihisa Nagatomo, Yoshihiko Atsuchi, Toshiro Nagoshi, Kazuhisa Sanno, Fumihiro Hoshino, Naoto Yokota, Masahiro Kameko, Toshifumi Tabuchi, Munesumi Ishizawa, Yoshitake Fujiura, Daisuke Ikeda, Taku Seto, Tetsu Iwao, Norio Ishioka, Kohei Nii, Koichi Oshiro, Keizo Tsuchida, Yutaka Hatori, Motoshi Takeuchi, Hiroto Takezawa, Shinjiro Nagano, Masaaki Iwaki, Yuichiro Nakamura, Naomasa Miyamoto, Toshifumi Taguchi, Ko Ashida, Naoto Yoshizawa, Jun Agata, Seishiro Matsukawa, Osamu Arasaki, Tetsuji Shinjo, Shuji Fukuoka, Hirofumi Murakami, Kazuya Mishima, Mamoru Manita, Yoshiki Hata, Ichiro Sakuma, Kotaro Obunai, Ichiro Takamura, Mitsuyuki Akutsu, Toshihide Unoki, Yoshinori Go, Makoto Ikemura, Shoji Morii, Shigeru Marusaki, Hideo Doi, Mitsuru Tanaka, Takaaki Kusumoto, Shigeo Kakinoki, Chiga Ogurusu, Kazuya Murata, Masaki Shimoyama, Masami Nakatsuka, Yutaka Kitami, Yoichi Nakamura, Hiroshi Oda, Rikimaru Oyama, Masato Ageta, Teruaki Mita, Kazuhiko Nagao, Takafumi Mito, Tsutomu Saito, Junichi Minami, Mitsunori Abe, Masako Fujii, Makoto Okawa, Tsuneo Fujito, Toshiya Taniguchi, Tenei Ko, Hiroshi Kubo, Mizuho Imamaki, Masahiro Akiyama, Takashi Ueda, Hironori Odakura, Masahiko Inagaki, Yoshiki Katsube, Atsuyuki Nakata, Shinobu Tomimoto, Mitsuhiro Shibuya, Masayuki Nakano, Kenichiro Ito, Masahiro Matsuta, Motoyuki Ishiguro, Taro Minagawa, Masamichi Wada, Hiroaki Mukawa, Masato Mizuguchi, Fumio Okuda Teruaki Kimura, Kuniaki Taga, Masaaki Techigawara, Morio Igarashi, Hiroshi Watanabe, Toshihiko Seo, Shinya Hiramitsu, Hiroaki Hosokawa, Mitsumoto Hoshiai, Michitaka Hibino, Koichi Miyagawa, Hideki Horie, Nobuyoshi Sugishita, Yukio Shiga, Akira Soma, Kazuo Neya, Tetsuro Yoshida, Kunio Akahane, Sen Adachi, Chiei Takanaka, Takashi Ueda, Saori Matsui, Hirofumi Kanda, Masanori Kaneko, Shiro Nagasaka, Atsushi Taguchi, Shuta Toru, Kazuyuki Saito, Akiko Miyashita, Hiroki Sasaguri, Jin Nariyama, Hiroyuki Miyamoto, Yusuke Nishida, Tatsuya Suga, Hiroki Nagata, Taketo Hatsuno, Takash Iwase, Kazuki Sato, Kazuya Kawai, Tomobumi Kotani, Tsuyoshi Tsuji, Hirosumi Sakai, Kiyoshi Nishino, Kenichi Ikeda, Kazuo Maeda, Tomohiro Shinozuka, Takeshi Inoue, Koichi Kawakami, Hiromichi Kitazumi, Tsutomu Takagi, Mamoru Hamaoka, Jisho Kojima, Akitoshi Sasaki, Yoshihiro Tsuchiya, Tetsuo Betsuyaku, Koji Higuchi, Masaaki Honda, Koichi Hasegawa, Takao Baba, Kazuaki Mineoi, Toshihiko Koeda, Kunihiko Hirasawa, Toshihide Kumazaki, Akira Nakagomi, Eiji Otaki, Takashi Shindo, Hiroyoshi Hirayama, Chikako Sugimoto, Takashi Yamagishi, Ichiro Mizuguchi, Kazunori Sezaki, Isamu Niwa, Ken Takenaka, Osamu Iiji, Koichi Taya, Hitoshi Kitazawa, Osamu Ueda, Hirokazu Kakuda, Takuya Ono, Seizo Oriso, Junya Kamata, Toshihiko Nanke, Itaru Maeda, Takashi Kawamoto, Yoshifusa Matsuura, Hiroki Teragawa, Yuichi Fujii, Shuichi Nomura, Tomohiro Ueda, Yasuyuki Maruyama, Kazuo Takei, Hajime Horie, Tetsutaro Kito, Hiroshi Asano, Koji Matsushita, Masaichi Nakamura, Takashi Washizuka, Tomoki Yoshida, Masato Sawano, Koji Matsushita, Shinichi Arima, Hidekazu Arai, Hisanori Shinohara, Hiroyuki Takai, Nobufusa Furukawa, Akira Ota, Kentaro Yamamoto, Kenji Aoki, Masahiko Abe, Rikiya Shinohe, Taku Yamamoto, Takeaki Kasai, Shunji Suzuki, Shu Suzuki, Kikuyo Takahashi, Nitaro Shibata, Masayuki Watanabe, Yosuke Nishihata, Yutaro Nishi,

Hiroyuki Niinuma, Yasuhiro Yokoyama, Hirotsugu Mitsuhashi, Ryo Nakazato, Takeaki Shirai, Yumi Shiina, Atsushi Mizuno, Toru Adachi, Taku Asano, Ikki Komatsu, Masahiro Yamazoe, Toru Arino, Masaki Okuyama, Tetsushi Wakiyama,Tomoko Kato, Yasuo Sasagawa, Takeshi Tana, Ayano Ishihara, Yoshihito Hayashi, Shinichi Hirota, Yukihiko Abe, Yoshihiro Saito, Hirohide Uchiyama, Hiroshi Takeda, Hiroshi Ono, Shuichi Tohyo, Naoto Hanazono, Seiichi Miyajima, Hisashi Shimono, Takuma Aoyama, Shusaku Miyata, Shigekiyo Takahashi, Takahisa Ido, Makoto Yamaura, Keita Suzuki, Yuto Kumai, Gen Tanabe, Takehiro Yamada, Yuka Kawada, Yasunobu Shozawa, Yawara Niijima, Osamu Murai, Osamu Murai, Hideko Inaba, Katsumasa Nomura, Masatsugu Nozoe, Kazuo Suzuki, Toshiyuki Furukawa, Toshihiko Shiraiwa, Nobuhisa Ito, Shunichi Nagai, Kiyoharu Sato, Shiro Nakahara, Yujin Shimoyama, Naoko Ohara, Teruhiko Kozuka, Hideaki Okita, Masato Endo, Tsutomu Goto, Makoto Hirose, Emiko Nagata, Noriyuki Nakanishi, Toshizumi Mori, Shuichi Seki, Katsuhiro Okamoto, Osamu Moriai, Yoko Emura, Tsuyoshi Fukuda, Haruhiko Date, Shuichi Kawakami, Sho Nagai, Yuya Ueyama, Tetsuro Fudo, Mitsuru Imaizumi, Takuo Ogawa, Shunsuke Take, Hideo Ikeda, Hiroaki Nishioka, Norihiko Sakamoto, Nobuo Wakaki, Masatake Abe, Junji Doiuchi, Tetsuya Kira, Masato Tada, Ken Tsuzaki, Naoya Miura, Yasuaki Fujisawa, Wataru Furumoto, Susumu Suzuki, Akinori Fujisawa, Ryosai Nakamura, Hiroyasu Komatsu, Rei Fujiki, Shuichi Kawano, Keijiro Nishizawa, Yoji Kato, Junya Azuma, Kiyoshi Yasui, Toshio Amano, Yasuhiro Sekine,Tatsuo Honzawa, Yuichiro Koshibu, Yasuhide Sakamoto, Yukihiro Seta, Shingo Miyaguchi, Kojuro Morishita, Yasuko Samejima, Toyoshi Sasaki, Fumiko Iseki, Toshiyuki Kobayashi, Hiroshi Kano, Jaeyoung Kim, Hiroshi Yamaguchi, Yoichi Takagi, Yoko Onuki Pearce, Yasuyuki Suzuki, Takayuki Fukui, Toru Nakayama, Hideaki Kanai, Yoshiyuki Kawano, Tetsuji Ino, Hironori Miyoshi, Yasufumi Miyamoto, Masahito Shigekiyo, Shimato Ono, Yoshiyuki Kawano, Yutaka Okamoto, Satoshi Ubukata, Kojiro Kodera, Tatsuo Oriuchi, Naoki Matsumoto, Koichi Inagaki, Atsushi Iseki, Tomohiro Yoshida, Toshihiro Goda, Tsukasa Katsuki, Atsushi Sato, Etsuo Mori, Toshio Tsubokura, Hiroshi Shudo, Shunichi Fujimoto, Tomohiro Katsuya, Yoshiyuki Furukawa, Hiroshi Hosokawa, Jun Narumi, Kiichiro Yamamoto, Masaki Owari, Takuya Inakura, Takafumi Anno, Kazuyuki Shirakawa.

Singapore

Chi Keong Ching, Sandra Thng, Eric Lim, Yap Pei Wen, Toon Wei Lim, Ying Ming Lee, Winnie Sia, Vivian Fong, David Foo, Haiyan Li, Ong See Peng, Tristen Ng, Kelvin Wong, Siew Yoon Yap, Dan Dan Chen, Geraldine Lim, Colin Yeo, Tan Yuyang, Zhi Zi Tan, Wan Tin Lim, Fazlur Rehman Jaufeerally.

South Korea

Seil Oh, JiHyun Sim, Hye Jin Song, Seungyun Lee, Tae-Eun Kim, HyunJu Yu, Hui Nam Park, Mi Suk Lee, Sung Yon Shim, So Hee Joo, Youngmi Park, Mihyang Park, Woo-Shik Kim, Eunjung Hwang, Eunsun Heo, Jin-bae Kim, Jung A Kim, HyeYoung Lee, Sunjoo Kim, Inae Han, Hiyan Ju Kim, Sung-Won Jang, Dong Bin Kim, SoYoung Yun, Dong Joon Kim, JinHee An, JiSuk Kwon, Dae Hyeok Kim and Kyung Mi Yoo, Jun Kim, Sang Eun Lee, Jeongsu Kim, DongRyeol Ryu, KyoungOk Her, Eun Hee Choi, Jaemin Shim, Sang Weon Park, Young Ran Jeon, JinYoung Ahn, Gyeong Hui Lee, Hye-Gyeong Jeon, Woo Jin Chi, Dae-Kyeong Kim, Seula Ye, Min Sun Ha, Sun Min Lee, Jin Hee Lee, Kyung Jin Lee, Yang-I Kim, Dong Ju Choi, Eun Hee Lee, Hee Kyung Choi, Woo Yeon Lee, EunJi Yoon, Yong Seog Oh, Yun Suk Son, Sae Rom Jeon, In-Ae Jung, Sukyung Park, Myeong-Chan Cho, Chung Suk Lee, Hack-Lyoung Kim, Sang Hyun Kim, Sang Hee Kim, EuMi Jang, Miseon Lim, Kyung Lee, Jiyeon Lee, Bo Ram Park, Hui-Kyung Jeon, Gyeong Hui Lee, Ki Tae Kim, Dong-Gu Shin, Seoyeon Jang, Jung-Min Kim, Kyung Hui Yim, Sang Weon Park, Jin Sik Park, Bomi Kami, Ju-Hee Ahn, Jae Seok Park, Jung Joo Ahn, Hoon Ki Park, Hee Suk Choi, Sang-Jin Han, Nari Ryu, NaYoung Kwon, Eun-Joo Choi, Jung Hoon Sung, JiSun Kang, So-Young Seo, HyeKyoung Wi, Hyung-Wook Park, JiHyeun

Park, Mi Ra Kim, Jeong-Gwan Cho, JiSeon Kim, Haeim Kim, Gi-Byoung Nam, Sulhee Lee, Keun Hye Lee, Young Keun On, Sehyun Kim, SoYoung Jo, Seung Hyun Lee, Hoejeoung Park, Minsuk Rhue, Min Joo Seo, Hong Euy Lim, Sang-Min Lee, SeongWook Hwang, Jae-Jin Kwak, Hyeji Bae, HyeYoung Kye, JaeJin Kwak, Tae-Joon Cha, Eun A Choi, In Kyoung Noh, Taek Jong Hong, JaeHyun Park, JaeKyung Kim, Jeong Hee Moon, Ro Woon Lee, Kyeonghwa Hyun, Seong Hoon Park, Mi-Young Lee, Jung Han Yoon, Jung Ja Woo, Hye-suk Jang, Sunhui Han, AReum Park, Won Sook Son, Hae Jung An, Nam-Ho Kim, Hae Kyeong Jeong, Ae Lee Baek, Kee-Sik Kim, Sung-Guk Kim, Mal-soon Park, Mi Sun Kim, JuHee Kim, Byung Chun Jung, Jung Sook Kim, Gyo-Seung Hwang, Doo Lae Lee, Kye Ryun Lee, Ji Sun Kang, Joo Yeon Yoo, Young Mi Sohn, Chong-Jin Kim, Hye-Young Lee, Dae Hyeok Kim, Kyung Mi Yoo.

Thailand

Sakda Rungaramsin, Riam Inphontan, Pimolwan Phunpinyosak, Peerapat Katekang-plu, Piyanuch Sukklad, Porames Khunrong, Thanita Bunyapipat, Suree Yawila, Wanwarang Wongcharoen, Arintaya Phrommintikul, Siriluck Gunaparn, Pinij Kaew-suwanna, Thida Chaipermkul, Khanchai Siriwattana, Theewarut Suttana, Ketsanee Khunkong, Waraporn Tiyanon, Thoranis Chantrarat, Panthipa Bamungpong, Supalerk Pattanaprichakul, Usa Kitmapawanont, Khanchit Likittanasombat, Bandit Na-ratreekoon, Kolltis Pongmorakot, Somluck Ninwaranon, Wacharin Suebjaksing, Doungrat Cholsaringkarl, Warangkana Boonyapisit, Napawan Pornnimitthum, Satchana Pumprueg, Sirichai Cheewatanakornkul, Ratchanee Sirichai, Arthiya Sriwichian, Treechada Wisaratapong, Songkwan Silaruks, Benjaporn Silaruks, Kittisak Sawanyaw-isuth, Pisit Hutayanon, Siripan Hongsuppinyo, Oranong Thangpet, Seksan Cha-wanadelert, Siriwan Ratchasikaew, Chuleerat Kongsin, Pairoj Chattranukulchai, Ru-deewan Khattaroek, Yongkasem Vorasettakarnkij, Boonsert Chatlaong, Yingsak Santana-korn, Khompiya Kanokphatcharakun, Piya Mongkolwongroj, Janwamol Phangyota, At-thakorn Wutthimanop, Thanawan Chaiyapong, Sasivimon Jai-Aue, Kanchana Sanit, Wat-tana Wongtheptien, Ongkarn Komson, Thanakorn Laksomya, Ampai Tangsirira, Suthasinee Poomiphol, Prathana Anekpunyakul.

Turkey

Armagan Altun, Elif Yildrim, Hatice Aysu Ocal, Didar Aslan, Aycan Arslan, Ozge Ayar, Ali Aydinlar, Hulya Saribocek, Gizem Yavasla, Ozlem Aktas, Nilay Gungor, Ram-azan Topsakal, Ibrahim Ozdogru, Leyla Kasa, Emine Yilmaz, Muruvvet Buyukpapuc, Mustafa Fehmi Bireciklioglu, Meryem Boyraz, Zeki Ongen, Fuat Polat, Elif Yilirim, Duygu Genc, Baris Ikitimur, Esra Kozig, Sinem Caliskan, Irem Ermec, Busra Gez, Fatih Koyun-cuoglu, Gamze Avinc, Sadik Acikel, Ekrem Yeter, Ahmet Akyel, Mehmet Ali Felekoglu, Asel Canbolat, Deniz Alpay, Duygu Ozukaya, Yonca Kanal, Ayla Behnejad Kazancik, Fa-ruk Yilmaz, Tilbe Kundak, Durmus Yildiray Sahin, Murat Cayli, Hasan Koca, Funda Balci, Hazal Sag, Taner Seker, Alaa Quisi, Berfu Dincyurek, Ilay Cevik, Yasemin Tuccar, Esra Sunduz Yigittekin, Ozcan Yilmaz, Dilek Zorlu, Elvan Ozcelik, Cansu Kiris, Mehmet Bir-han Yilmaz, Funda Aras, Osman Beton, Hasan Pekdemir, Merve Zengin, Refah Karatas, Murdem Keten, Mesut Demir, Vildan Yuksekdag, Aziz Celik, Hatice Rahim, Didem Uzun Alkan, Gulsum Daglik, Murat Sucu, Erdi Bilecen, Selin Budeyri, Sinan Bilecen, Levent Sa-hiner, Hikmet Yorgun, Ali Oto, Duygu Ozukaya, Murat Ersanli, Deniz Koca, Ali Oto, Ser-can Okutucu, Begum Yetis Sayin, Deniz Koca, Asel Conbolat, Deniz Alpay, Duygu Ozu-kaya, Mine Caglar, Sebnem Sebnem, Murat Ersanli, Sakine Sakiz, Esra Kozig, Sinem Cal-iskan, Irem Ermec, Busra Gez, Faith Koyuncuoglu, Gamze Avinc, Ertugrul Okuyan, Ekrem Bilal Karaayvaz, Rifat Yildirim, Sinan Varol, Fatih Kizkapan, Sakine Sakiz, Esra Kozig, Sinem Caliskan, Irem Emrec, Busra Gez, Faith Koyuncuoglu, Gamze Avinc, Dur-sun Aras, Didem Civit, Tuba Gulce Dodurga, Asel Canbolat, Deniz Alpay, Duygu Ozu-kaya, Yonca Kanal.

Argentina

Florencia Rolandi, Natalia Vensentini, Vanesa Hansen, Adrian Cesar Ingaramo, Gustavo Alberto Ingaramo, Andres Javier Kleiban, Mariana Zillo, Maria Eugenia Yunis, Martin Racca, Carola Ricotti, Luciana Bergesio, Osvaldo Jose Angel Costamagna, Gustavo Alberto Sambadaro, Vanina Fernandez Caputi, Fernando Sokn, Jorge Tronge, Andrea Alvarez D'Amelio, Clara Buzzetti, Pablo Omar Schygiel, Hector Luciardi, Sofia Graciela Berman, Pablo Dragotto, Felicitas Fernandez Voena, Andres Javier Kleiban, Maria de los Milagros Mercedes Had, Karina Shatski, Nestor Centurion, Elena Emilia Piccirilli, Rodolfo Andres Ahuad Guerrero, Andrea Alvarez D'Amelio, Vanina Campisi, Mariano Fanuele, Agustina Ahuad Calvelo, Leonel Adalberto Di Paola, Flavio Javier Zurbrigk, Anabela Martinelli, Ricardo Dario Dran, Carlos Hector Gimenez, Javier Egido, Alicia Sossich, Fernando Colombo Berra, Cintia Schenkel, Melina Salinger, Luciana Montoya, Matias Jose Fosco, Enrique Pablo Gurfinkel, Branco Mautner, Simona Malengo, Emilio Daniel Alaguibe, Lucia Campo, Victor Alfredo Sinisi, Luis Rodolfo Cartasegna, Andrea Santoro, Maria Fernanda Alzogaray, Viviana Novas, Jesica Florencia Tinto, Oscar Gomez Vilamajo, Romina Cabrini, Javier Matkovich, Valentina Garate, Maria Eugenia Said Palladino, Jose Luis Ramos, Fabian Diez, Pablo Dragotto, Laura Susana Sanziani, Cintia Martinelli, Amaru Lopez, Sonia Sassone, Laura Maffei, Miguel Angel Vallejo, Marcelo Yantorno, Lucas Tonelli, Roxana Del Valle Martinez, Maria Goicoechea, Micaela Vallejo, Jesica Pontoriero, Rocio Munguia, Maria Levantini, Flavia Luciana Cilenti, Graciela Beatriz Perez Prados, Marina Ingratta, Gerardo Zapata, Anibal Agustin Damonte, Luciana Arias, Alejandro Meirino, Diego Conde, Jorge Atilio Belardi, Ignacio Nicolas De Urquiza, Alexandra Navarro, Juan Pablo Costabel,Guillermo Giacomi, Maria Paula Giacomi, Ana Laura Tufare, Ramon Carrizo, María Florencia Edén, Alberto Alfredo Fernandez, Roberto Nicolás Potito, Silvina Borchowiec, Tatiana Bruno, Adrian Demetrio Hrabar, Jorgelina Casala, Claudia Funosas, Ana Lucia Cappi, Mario Alberto Berli, Paula Berli, Florencia Berli Milagros, Leandro Thomas, Mauricio Priotti, Germán Maehara, Fabian Ferroni, Celso Arabetti, Mariana Foa Torres, Maria Jose Izaguirre.

Brazil

Dário Celestino Sobral Filho, Libania Ferreira, Tricia Souto Cysneiro, Eveline Lustosa, Jefferson Jaber, Luis Soares, Isabel Santos, Luciana Vidal Armaganijan, Dikran Armaganijan, Simone Barroso, Kleber Serafim, Maria Isabel Del Monaco, Costantino Roberto Frack Costantini, José Rocha Faria Neto, Carolina Stoll, Danilo Gonçalves Barroso, Costantino Costantini Ortiz, Daniele Komar, André Steffens, Camila Fonseca, Felipe da Silva Paulitsch, Weimar Kunz Sebba Barroso de Souzaem, Lohana Borges Queiroz, João David de Souza Neto, Dafne Lopes, José Márcio Ribeiro, Marcelo Silveira Teixeira Denise Guanaes, Paulo Ross, Verena Araujo, Luana Herek, Leonardo Pires, Oscar Pereira Dutra, Silvia Poletti, Rafael de March Ronsoni, Daniel Moreira, Vera Lucia Pereira, José Carlos Moura Jorge, Leonardo Spolaor, Cristaine Dias, Mariana Rodrigues Pius, Juliane Woehl, Adalberto Menezes Lorga Filho, Eduardo Palmegiani, Priscila Fabri, Paula Galeazzi, Thamyres Santini Arroio Cruz, Clotildes Queirantes, Luiz Bodanese, Tulio Ruaro Reichert, Fernanda Chieza, Rosa Vieira Homem, Ellen Hettwer Magedanz, Marcelo Westerlund Montera, Roberta Perreira, Carlos Henrique Del Carlo, Eliane Fernandes, Gabriela Melo, Esther Coste, Maria Fernanda Correia, Cristiano Pisani, Muhieddine Omar Chokr, Hurgo Bellotti Lopes, Ricardo Clemente Mingireanov, Talita Barbosa, Selma Cristina Quaia Fortunato, Elaine Fernandes, Jamil Abdalla Saad, Thiago da Rocha Rodrigues, Barabara Silva, Crystielle Linhares, Maria Celia Marinho, Tânia Félix Lorenzato Fonseca, Eduardo Belisario Falchetto, Fernando Augusto Alves da Costa, Raquel Franchin Ferraz, Renato Lopes, Anelise Kawakami, Erika Watanabe, Mayara Vioto Valois, Gilson Roberto de Araújo, Lenisa Vila Boas, Euler Roberto Manenti, Máurer Pereira Martins, Adriana Silva, Priscila Aparecida Lodi, Aline Borba, Laisa Borrges Ferreira, Anderson Lacerda dos Reis, Nurma Ramos Pereira, Aline de Cassia Vieira dos Santos, Mariana Moraes Pontalti, Alexandre Guerreiro, Ana Paula Macagnan, Livia

Pereira, Patricia Ely Pizzato, Milene Santos, Rafael Luiz Rech, Jose Francisco Kerr Saraiva, Larissa Trama, Carla Vicente, Camila Ormundo, Midia Costa, Tatiana Siqueira, Marina Caporale, Talita Silva, João Carlos Ferreira Braga, Lilian Stefanie da Silva, Daiane Santos, Alexandre Rodrigues, Fábio Villaça Guimarães Filho, Alexandre Negri, Paulo Gottardo, Carlos Moncada, Marcos Vinicius Seroqui, Dalton Precoma, Lucas Geralde, Cibelle Precoma, Thiago Felipe dos Santos, Cristina Paula Corrêa, Luiz Gustavo Vieira Torres, Fernando Roquette, Gilmar Reis, Ludmila Carvalho, Roberto Álvaro Ramos Filho, Caroline Nanzer Vital, José Da Silveira, Rodrigo Pavani, Estêvão Lanna Figueiredo, Bruna Edilena Paulino Azevedo, Beatriz Quirino, Gustavo Fonseca Werner, Roberto Vieira Botelho, Iara Resende, Cláudio Munhoz da Fontoura Tavares, Rogerio Tadeu Tumelero, Gilmar Junior, Norberto Toazza Duda, Leticia Mortari, Melissa Mazzoni, Helius Carlos Finimundi, Silvia Oss Emmer, Fábio Eduardo Camazzola, Edimar Daros, Juliana Anghinoni Andretta, Bruna Nadin, Cibelle Precoma, Sara Cardoso Boscato, Adriano Kochi, Diego Chemello, Juliani da dos Reis, Sabrina Guizzardi, Priscila Agliardi, Fernanda Igansi, César Cássio Broilo França, Jakeline Alves de Oliveira Gomes, Ana Paula Drummond Wainstein, Edmar Geraldo Ribeiro, Guido Bernardo Aranha Rosito, Heveline Roesch, Larissa Pacheco, Jordana Alba, Tatiana Vargas, Andressa Degen, Diego Alcoba, João Batista de Moura Xavier Moraes Junior, Fernanda Freire, Sheylla Ribeiro, Maria Aparecida Torres de Lacerda, Rogério Tadeu Tumelero, Gilmar Junior, Norberto Toazza Duda, Melissa Mazzoni, Claudio Munhoz da Fontoura Tavares, Leticia Mortari, Lilia Maia, Osvaldo Lourenço da Silva Júnior, Daniele Cristine Esteves, Osana Maria Coelho Costa Mouco, Maria Angelica Benez Teixeira Lemos, Nadielly Codonho Góes, Marcelo Arruda Nakazone, Roberto Simões de Almeida, Lucas Frare, Bruna Unterkircher, Ney Carter do Carmo Borges, Fernanda Jannuzzi, Marco Antonio Dias, Luís Gustavo Gomes Ferreira, Etineia Gomes Lino, Arthur Freitas, Tatiane Viana, Manoel Lucas Correa e Silva, Angela Lopes.

Chile

Ramón Corbalán, Ivonne Padilla, Jara Carmen, Benjamin Aleck Joseh Stockins Fernandez, Carola Lara, Humberto Montecinos, Yessica Campisto, Fernando Lanas, Ana Rebolledo, Elda Molina, Martín Larico Gómez, Carlos Astudillo, Margarita Vergara, Carlos Conejeros, Patricio Marin Cuevas, Alejandro Forero, Claudio Bugueño Gutiérrez, Patricia Cortes, Juan Aguilar, Sergio Potthoff Cardenas, Nelly Garcia Dominguez, German Eggers, Jessica Munoz Oyarzon, Cesar Houzvic, Veronica Olguin, Carlos Rey, Germán Arriagada, Claudio Villan Araneda, Gustavo Charme Vilches, Ximena Reyes Alvarez.

Mexico

Carlos Jerjes Sanchez Diaz, Andres Gerardo Ortiz López, Hector David Rodriguez Flores, Claudia Atilano Arias, Jesus Jaime Illescas Diaz, Maria Ruiz Cornejo, Maricela Vidrio Velazquez, Maria Esther Garcia Muñoz, Marissa Gonzalez, Raul Leal Cantu, Pilar Ocampo, Conne Lizbeth Gonzalez Garcia, Conne Lizbeth Gonzalez Garcia, Maria Guadalupe Ramos Zavala, Sara Pascoe González, Ricardo Cabrera Jardines, Nilda Espinola Zavaleta, Victor Roa Castro, Salome Altamirano Bellorin, Enrique Lopez Rosas, Isis Lizeth Alarcon Zaragoza, Sukey Ochoa Aybar, Lucas Solis Morales, Guillermo Antonio Llamas Esperón, Irma Espinosa, Ana Ramirez Ibarra, Gabriela Romero Cardona, Edgar Eduardo Barcenas Rodriguez, Edgar Eduardo Barcenas Rodriguez, Hector Alejandro Martinez Resendiz, Maria Elena Felix Hernandez, Gerardo Pozas, Priscila Guajardo, Ernesto Cardona Muñoz, Sandra Hernandez Gonzalez, Norberto Matadamas Hernandez, Maria Candelaria Mancilla Ortiz, Adolfo Leyva Rendon, Adriana Ortiz, Norberto Garcia Hernandez, Rafael Trujillo Cortes, Manuel de los Rios Ibarra, Olivia Elena Espinoza Lopez, Luis Gerardo Gonzalez Salas, Nora Mendoza, Luis Ramon Virgen Carrillo, Oscar Garcia Garciia, Jose Vega Gonzalez, Humberto Jimenez, Aldo Guasco Herrera, David Lopez Villezca, Carlos Hernandez Herrera, Daniel Martinez Vasquez, Karla Yaneth Arizpe Aguilar, Aline Cecilia Meza Ramos, Juan Jose Lopez Prieto,

Rodolfo Gaona Rodriguez, Patricia Padilla Macias, Efrain Villeda Espinosa, Angelica Villeda, Artuto Gil Sanchez Aguilar, Margarita Abreo, Miriam Chavarria, David Flores Martinez, Jose Velasco Barcena, Omar Fierro Fierro, Maria de la Merced Velazquez Quintana, Jorge Morales, Anais Rascon, Luz Eighty Garcia Prieto, Rosalva Avena, Lesly Facio, Ignacio Rodriguez Briones, Bernardo Vargas Berrueta, Monica Ramos Gonzalez, Irene Elizabeth Lira González, Jose Luis Leiva Pons, Jorge Carrillo Calvillo, Natalia Nikitina, Teresa Izquierdo, Humberto Alvarez Lopez, Gabriela Santana, Rafael Olvera Ruiz, Carlos Gerardo Cantu Brito, Fernando Flores Silva, Eduardo Julian Jose Roberto Chuquiure Valenzuela, Roxana Reyes Sanchez, Ricardo Alvarado Ruiz, Lidia Martínez Lares, Paulina Sida Perez, Alberto Esteban Bazzoni Ruiz, Erika Orpinel Almanza, Karla Daniela Monreal de Luna, Aglaé Romo Cerda, Juan Carlos Ubillo Davila, Sandra Roccio Fuentes Ortiz, Rocio Guadalupe Contreras Corona Alejandro Maximiliano Bacelos Lopez, Jose Luis Recio Gonzalez, Oscar Martin Lopez Ruiz, Fatima Perez, Luis Alberto Martinez Cruz, Julio Alberto Perez Sanchez, Nayeli Soto, Eduardo Montiel, Norma Juarez Garcia, Keren Sharon Perez Mendoza, Oscar Nandayapa Flores, Roberto Arriaga Nava, Patricia Padilla Macias, Jesus David Morales Cerda, Antonio Salinas, Sergio Piña Toledano, Euince Aide Fernandez Zuñiga, Pedro Fajardo Campos, Daniela Cienfuegos, Karina Godoy, Silvia Verdugo Dojaquez, Elena Claudia Mora Respardo, Pablo Alfonso Madero Ayala, Marittza Arasely Uribe Rios, Guadalupe Susan Ramos Garcia, Pedro Fajardo Garcia, Adriana Garcia Mejia, Patricia Ortiz, Glenda Hernandez, Irene Ortega, Mario Benavides Gonzalez, Jose Esparza Negrete, Carlos Rivera Ramos.

Austria

Marianne Brodmann, Thomas Gary, Claudia Wöhrer, Claus Hagn, Johannes Foechterle, Johannes Foechterle, Heinz Drexel, Karl-Martin Ebner, Abdurahman Said, Daniela Zanolin, Claudia Stöcklöcker, Alexandra Schuler, Alexander Vonbank, Susanne Wäger, Peter Schwerzler, Helga Winkler, Kurt Huber, Claudia Wegmayr, Ioannis Tentzeris, Brigit Vogel, Johannes Riedl, Andrea Podczeck-Schweighofer, Fritz Freihoff, Thomas Priesnitz, Michael Winkler, Roland Breier, Bruno Schneeweiss, Bernadette Bruckner, Thomas Mark, Adrian Mirtl, Alfons Gegenhuber, Wilfried Lang, Agnes Lischka-Lindner, Sabine Eichinger-Hasenauer, Lisbeth Eischer, Peter Kaserer, Josef Sykora, Heribert Rasch, Bernhard Strohmer, Uta Hoppe, Lynne Hinterbuchner, Erika Prinz.

Belgium

Luc Capiau, Hilde Capiau, Farah Banaeian, Geert Vervoort, Ellen Deweerd, Erwin Raymenants, Oscar Semeraro, Muriel Delvigne, Tom Vydt, Hans Rombouts, Chris Scheurwegs, Ellen Potoms, Inez Mestdagh, Sofie Drieghe, Bart Wollaert, Benjamin Scott, Tim Weyn, Emile Keyzer, Marleen Bogaert, Martine Oreglia, Inge Joris, Dirk Denie, Christine Jacobs, Ingrid Develter, Frank Cools, Dieter De Cleen, Steven Hellemans, Johan Salembier, Jef Verheyen, Walter Smolders, Nancy Simons, Veerle Thyssen, Geert Hollanders, Jan Vercammen, Veerle Soufflet, Jan De Keyser, Dries De Cock, Melissa de Vos, Dirk Faes, Bert Vanhauwaert, Dirk Van Lier, Cathy Kuppens, Ann Vandorpe, Yohan Balthazar, Anne Christine Billiaux, Marc Delforge, Patrick Maréchal, Francoise Gits, Olivier Xhaet, Fabien Dormal, Harry Striekwold, Dirk Vandenbroeck, John Thoeng, Kurt Hermans, Hans Vandekerckhove, Chantal Smessaert, Hugues Verloove, Katarina Van Beeumen, Jan Nimmegeers, Christophe Borin, Frauke Gorre, An-Kristin Ascoop, Nancy de Weerdt, Georges Mairesse, Monique Raepers, Wim Anné, Emma Vanhalst, Peter Pollet, Anne Dewispelaere, Florian Demuynck, Martin De Meyer, Tine Casier, Ivan Blankoff, Veronica Piamonte, Asuncion Conde Y Bolado, Michel Beutels, Peter Vandenbossche, Sophie Deckers, Lisa Helvasto, Stefan Verstraete, Karine Pieters, Flor Kerkhof, Ann Dhondt, Peter Vandergoten, Philippe Purnode, Joseph Richa, Severine Tahon, Pascal Godart, Anna-Maria Barbuto, Marianne Blockmans, Tim Boussy, David Derthoo, Christiaan De Niel, Van Hulle Tim, Hannelore Van Eeckhoutte, Nele Bouckaert, Marlies De Coninck, Philippe

Desfontaines, Gabriella Tincani, Alex Heyse, Charlotte Vantomme, Becker Alzand, Frederik Van Durme, Isabelle Stockman, Marloes Everaert, Joeri Voet, Lies Vergauwen, Martine Lauwers, Yasmina Steyaert, Axel De Wolf, Chris Brike.

Czech Republic

Jan Bultas, Hana Lubanda, Milan Zidek, Eva Zidkova, Alena Lorenzova, Petr Jansky, Petra Antonova, Jiri Zika, Jana Golova, Alexandra Lindourkova, Jaromir Chlumsky, Petr Potuznik, Renata Kratochvilova, Rudolf Spacek, Lucie Mahdalikova, Lucie Bockova, Adam Sulc, Lenka Dastychova, Vilma Machova, Ondrej Ludka, Alexandra Ludkova, Josef Olsr, Daniel Michalik, Lubos Kotik, Marketa Lajnerova, Blazej Racz, Michaela Horejsi, Lenka Janska, Tomas Drasnar, Richard Ferkl, Jan Hubac and Vera Hubacova, Ilja Kotik, Jaroslava Novakova, Petr Krca, Eva Krcova, Zdenek Monhart, Eva Prochazkova, Maria Majerníkova, Hana Burianova, Pavel Hanzelka, Ondrej Jerabek, Martina Valtova, Iva Kopeckova, Jana Pisova, Lenka Chmelickova, Iveta Petrova, Lenka Palubova, Vratislav Dedek and Lucie Capova, Michaela Honkova, Jakub Honek, Petr Podrazil, Petr Reichert, Lenka Zizkova, Jindrich Spinar, Ruzena Labrova, Miroslav Novak, Jiri Vitovec, Veronika Vyhlidalova, Jolana Lipoldova, Vaclav Durdil, Alexandra Lindourkova, Lucie Riedlbauchova, Katarina Plocova, Martin Barsovsky, Jaroslav Sulitka, Jiri Lastuvka, Sarka Smetankova, Lenka Zizkova, Jakub Sveceny.

Denmark

Jørn Nielsen, Steen Husted, Marianne Leth, Helena Dominguez, Henrik Nielsen, Ulrik Hintze, Anne-Mette Oksbjerg Svenningsen, Joan Hummelshøj, Lene Margrethe Tanggaard, Annette Nygaard, Søren Rasmussen, Arne Bremmelgaard, Christina Ellervik, John Markenvard, Kirsten Vesterager, Jan Børger, Jorgen Solgaard, Ebbe Eriksen, Peter Simonsen, Jakob Tilma, Thomas Løkkegaard, Michael Bruun, Jacob Mertz, Morten Schou, Krud Skagen, Helena Dominguez, Annie Therkelsen, Helena Dominguez, Jesper Hansen, Anita Meier, Ilan Raymond, Dorte Raae, Michael Olsen, Magnus Jensen, Natasaha Roseva-Nielsen, Lise Jensen, Therese Bang-Hansen.

Finland

Pekka Raatikainen, Kati Helleharju, Hannaleena Nappila, Virpi Palomak, Olli Arola, Juhani Koistinen, Tuija Vasankari, Carmela Viitanen.

France

Franck Paganelli, Didier Sanchez, Pauline Armangau, Deborah Setbon, Audrey Morioni, Floriane Robin, Joël Ohayon, Frédéric Casassus, Stéphane Lafitte, Eléonore Casassus, Elodie Ducasse, Jean-Yves Le Heuzey, Michel Galinier, Nathalie Rosolin, Yannick Gottwalles, Franck Paganelli, Didier Sanchez, Audrey Morioni, Floriane Robin, Deborah Setbon, Pauline Armangau, Philippe Loiselet, Jean-Joseph Muller, Mohamed Bassel Koujan, André Marquand, Sylvain Destrac, Olivier Piot, Nicolas Delarche, Josette Couleru, Xavier Giry, Christine Murguet-Badia, Anne Colomes, Jean-Pierre Cebron, Nathalie Decarsin, Maxime Guenoun, Dominique Guedj-Meynier, Lokesh A G, Daniel Galley, Christine Beltra, Mathieu Zuber, Anne Bonnetain, Pierre Amarenco, Agnes Kemmel, Emmanuel Ellie, James Kadouch, Pierre-Yves Fournie, Jean-Pierre Huberman, Nestor Lemaire, Gilles Rodier, Xavier Vandamme, Igor Sibon, Sylvain Ledure, Sabrina Debruxelles, Camille Girollet, Stephane Olindo, Mathilde Poli, Pauline Renou, Sharmila Sagnier, Nathalie Heyvang, Ledure Sylvain, Jean-Philippe Neau, Marie Hélène Mahagne, Alain Suissa, Antoine Mielot, Marc Bonnefoy, Jean-Baptiste Churet, Vincent Navarre, Frederic Sellem, Gilles Monniot, Jean-Paul Boyes, Bernard Doucet, Michel Martelet, Désiré Obadia, Bernard Crousillat, Joseph Mouallem, Etienne Bearez, Corinne Lepot, Jean Philippe Brugnaux, Alain Fedorowsky, Pierre Nazeyrollas, Jean-Baptiste Berneau, Angeline Barreau, Aurélie Pons, Estelle Corrihons, Frédéric Chemin, Margaux Treuil-Peraldi, Elodie Ducasse, Nadege Ansoult, Caroline Moreau.

Germany

Sebastien Schellong, Andreas Ulbrich, Thomas Wolf, Carsen Mueller, Johannes Ullrich, Roswitha Frommhold, Kerstin Spranger, Lutz Pomper, Birgit Voigts, Slyvia Weichelt, Juliane Gehre, Mario Hahn, Harald Darius, Friederike Girke, Katja Schirmer, Sandra Gaulke, Thomas Braun, Tanja Toennishoff, Roya Sartipi, Viola Jaerisch, Elke Luczak, Carsten Meincke, Stefan Sommer, Astrid Maselli,: Georg Koeniger, Andreas Kopf, Uwe Gerbaulet, Bernd-Thomas Kellner, Thomas Schaefer, Gabriele Jass, Sabrina Diez, Jan Purr, Ute Weiser, Enno Eißfeller, Karla Kirchner-Volker, Heinz-Dieter Zauzig, Peter Riegel, Christoph Axthelm, Juergen Wurziger, Anja Quietzsch, Janet Geyer, Hartmut Hohensee, Cornelia Fritz, Adrienne Heuer, Benjamin Schaefer, Gerd-Ulrich Heinz, Afra Heinz, Holger Menke, Andreas Pustelnik, Sabine Pustelnik, Stefan Zutz, Andrea Talkenberger, Wolfgang Eder, Zuzana Babjakova, Guenter Rehling, Dirk Glatzel, Norbert Ludwig, Petra Sandow, Henning Wiswedel, Cosmas Wildenauer, Wolfgang Wildenauer, Svetlana Wildenauer, Steffen Schoen, Christoph Reichelt, Steffi Turbanisch, Romy Schoene, Stephanie Zincke, Marian Christoph, Toralf Schwarz, Heike Riessbeck, Adyeri Babyesiza, Regina Voelkel-Babyesiza, Maximilian Kropp, Sylvia Simon, Hans-Hermann Zimny, Marianne Hintze, Hildegard Floegel, Friedhelm Kahl, Andreas Caspar, Sabine Omankowsky, Torsten Laessig, Hermann-Josef Hartmann, Diana Amrhein, Kartin Tauber, Ramona Heeg, Gunter Lehmann, Aileen Grytzmann, Hans-Walter Bindig, Katharina Bindig, Gunter Hergdt, Susanne Hergdt, Dietrich Reimer, Cornelia Reimer, Joachim Hauk, Christl Hubrich, Holger Michel, Werner Erdle, Wilfried Dorsch, Aneta Tetlak, Janna Dshabrailov, Karl-Albrecht Rapp, Reinhold Vormann, Daniela Nicolaus, Thomas Mueller, Peter Mayer, Uwe Horstmeier, Jan Zak, Volker Eissing, Gabriele Menken, Heinz Hey, Heinz Leuchtgens, Volker Lilienweiss, Heiner Mueller, Kruth Kolitsch, Anja Fleck, Katja Bergner, Christian Schubert, Herrmann Lauer, Thomas Buchner, Gunter Brauer, Diana Kroll, Susanne Kamin, Karsten Mueller, Gabriela Mueller, Sylvia Baumbach, Kerstin Schmidt, Muwafeg Abdel-Qader, Dagmar Mohrmann, Anke Krueger, Hans-Holger Ebert, Lisa Mann, Helen Mortan, Antje Richter, Gunter Stenzel, Romy Meinecke, Carsten Schwencke, Stefanie Boehme, Manfred Geiger, Detlef Mathey, Kerstin Flint, Sebastian Schellong, Thomas Wolf, Carsen Mueller, Lutz Pomper, Andreas Ulbrich, Juliane Gehre, Johannes Ullrich, Roswitha Frommhold, Kerstin Spranger, Birgit Voigts, Sylvia Weichelt, Mario Hahn; Universitaetsklinikum Ulm, Ulm, Germany: Peter Bernhardt, Dominik Buckert and Uta Dichristin, Laszlo Karolyi, Yvonne Nickstadt, Kartin Boehme, Christin Rade, Kerstin Bonin, Kerstin Mikes, Franziska Guenthe, Britta Sievers, Veselin Mitrovic, Wilhelm Haverkamp, Nesrin Nasser, Marika Saegebarth, Philipp Lacour, Paula Muenkler, Luis Weitbrecht, Katja Hubert, Jens-Uwe Roehnisch, Susanna Scharrer, Florian Busch, Katja Helgert, Vanessa Klein, Julainne Wettengel, Susanne Zeifele, Carola Ravenhorst, Maria Schuppe.

Hungary

Andras Papp, Andras Vertes, Gabor Szantai, Andras Matoltsy, Tunde Kiss, Nikosz Kanakaridisz, Zoltan Boda, Erno Kis, Jusztina Feil, Balazs Gaszner, Ferenc Juhasz, Zsuzsanna Hollo, Peter Jen, Gizella Juhasz, Zsolt Ples, Veronika Forgo, Szilvia Gergely, Petra Gombos, Sandor Kancz, Eszter Fulop, Zoltan Laszlo, Boglarka Vandrus, Zoltan Radics, Zsolt May, Fruzsina Sztanyik, Bela Merkely, Anna Hermecz, Marianna Szabo, Nora Sydo, Ebrahim Noori, Maria Hollósiné Kovács, Tamas Habon, Kalman Toth, Szilvia Kovacsne Levang, Peter Polgar, Attila Szilagyi, Gabriella Szalai, Sandor Vangel, Andras Nagy, Gabriella Engelthaler, Istvan Szombati, Gabor Bogye, Eszter Kiralyhazine Gyorke, Judit Ferenczi, David Gulyas, Emese Dohovits, Anette Toth, Reka Szalo, David Cseresznyek.

Italy

Giuliana Martini, Leone Maria Cristina, Attilia Pizzini, Eros Tiraferri, Rita Santoro, Sophie Testa, Cinzia Zecca, Giovanni Di Minno, Pasquale Ambrosino, Marco Moia, Simon Braham, Teresa Maria Caimi, Roberta Fritella, Daniela Piazzolla, Roberta Frittella, Elena Mollica Poeta, Stefania Brusorio, Maria Tessitori, Giancarlo Agnelli, Federica Macellari, Erika D'Agostini, Marta Fedele, Roberto Cappelli, Michele Voglino, Daniela Poli, Roberto Quintavalla, Piera Maria Ferrini, Franco Cosmi, Raffaele Fanelli, Raimondo Massaro, Carmela DArienzo, Vincenzo Oriana, Gaterina Latella, Gianluca Sottilota, Raffaele Reggio, Roberto Santi, Leonardo Pancaldi, Giulio Boggian, Raimondo De Cristofaro, Leonardo Di Gennaro, Giuliana Guazzaloca, Gualtiero Palareti, Luisa Salomone, Angelo De Blasio, Carlo Ciabatta, Jorge Salerno Uriate, Vincenzo Guerrieri, Benedetta Montanari, Alfredo Bianchi, Riccardo Gorla, Flavia Lillo, Franca Minetti, Cinzia Scarone, Enrico Maria Pogliani, Monica Carpenedo, Grzegorz Bilo, Dr. Luca Grappiolo, Michele Accogli, Simona Longo, Antonio Mariani, Nino Ciampani and Francesca Calcagnoli, Anna Patrignani, Mauro Feola, Arturo Raisaro, Katharina Granzow, Luciano Fattore, Cosimo Nave, Andrea Mauric, Fabrizio Germini, Giuliana Duranti, Gregorio Baglioni, Valerio Pannacci, Fulvio Forcignanò, Angelo Giombolini, Luca Tedeschi, Maria Settimi, Mario Berardi, Sergio Nicoli, Paolo Ricciarini, Roberto Nasorri, Antonio Argena, Pierdomenico Bossolasco, Bartolomeo Allasia, Paolo Ronchini, Claudio Bulla, Luciano Foppa, Alessandro Filippi, Maria Lusia Bottarelli, Antonino Tomasello, Filippo Tradati, Massimo Volpe, Maria D'Avino, Domenico Caruso,: Maria Grazia Bongiorni, Luca Segreti, Silva Severi, Marco Breschi, Alessandro Capucci, Federico Guerra, Kate Pozniak Graziela Rangel, Marta Marcinekova, Corrado Lodigiani, Elena Banfi,Veronica Pacetti, Enrico Salomone, Gaetano Serviddio, Rosanna Villani, Aurelio Lo Buglio, Claudio Tondo, Viviana Biagioli, Eleonora Russo, Giuseppe Ambrosio, Giuseppe Ciliberti, Ezio Mesolella, Paolo Golino, Giovanni Cimmino, Carmine Mazzone, Andrea Di Lenarda, Saverio Iacopino, Giuseppe Campagna Pierpaolo Occhilupo, Roberta Spirito.

The Netherlands

Hugo ten Cate, Marieke Pavlicic, Noordwest Ziekenhuisgroep, Alkmaar, J.H. Ruiter, Andreas Lucassen, H.C. Klomps, Henk Adriaansen, Mirjam Debordes, Jan Jaap van Putten, Maarten Bongaerts, Laurens Westerman, Jannie Boon, Tineke Kloosterman, Mathijs Pieterse, Misannia Boersma-Slootweg, Salcudean Tania, Stan Vergos, Kris Gorrebeeck, Coen van Guldener, Johannes Herrman, Femmy Bosman, Sara Dols, Laura Breukel, Jennifer Knaake, Sander Niehe, Elise Van Dongen Sandra Bruin, A.J.K. Roelse, S.H.K.Josien Krikken, Eileen Van Warners, Floor Geerlings, P.R. Nierop, Ingrid Danse, Nel Slingerland, Jurgen Akkerhuis, Sweder Van de poll, Marja van der Knaap, Irene Kort, Margaret Dirks, Pieter Hoogslag, J.H. Geertman, Agaath Stallinga-de Vos, Walter Hermans, Paul Melman, Tim van der Kley, Guido van Leeuwen, A. Pronk, Riny van de Loo, Jeanne de Graauw, B.E. Groenemeijer, Anastazia Jerzewski, E. M. Koomen, Suzanne Jansen, Rina Mulder, W. Terpstra, J.M.C. Van Hal, Anneke Grunewald, Cees Buiks, Paul Buiks, L.V.A. Boersma, Pim Tonino, Jur ten Berg Josine te Kaat, Wesley Jetten, Malu Kelderman, Judith De Graaf, Mike Bosschaert, Boudewijn Uppelschoten, Irene Vogel, Donia Mohamed, Madeleine van der Perk.

Norway

Ingrid Dominguez, Dan Atar, Bjørn Løvås, Trude Berge, Eivind Berge, Hege Claussen, Per Anton Sirnes, Vivi Nilsen, Erik Gjertsen, Torstein Hole, Rigmor Bøen, Silje Rasmussen, Knut Erga, Arne Hallaråker, Marie O'Donovan, Gunnar Skjelvan, Sissel Strand, Anders Østrem, Beraki Ghezai, Arne Svilaas, Kristin Ringdalen, Peter Christersson, Torbjørn Øien, Aase Jekthammer, Svein Høegh Henrichsen, Anne Berit Lensebraaten, Jan Erik Otterstad, Karin Ausen, Jan Berg-Johansen, Hege Antonsen.

Poland

Janina Stepinska, Elzbieta Kremis, Anna Konopka, Andrzej Gieroba, Malgorzata Biedrzycka, Lukasz Biedrzycki, Michal Ogorek, Aleksandra Szczepanska, Beata Wozakowska-Kaplon, Radoslaw Bartkowiak, Kamila Wesolowska, Rafal Bzymek, Elzbieta Jaskulska-Niedziela, Krystyna Loboz-Grudzien, Edyta Szuchnik, Magdalena Dziuba, Barbara Rzyczkowska, Maria Loboz-Rudnicka, Jaroslaw Kosior, Ewa Pawlik-Rak, Wieslaw Supinski, Anna Szulowska, Jerzy Bartnik, Jerzy Kuzniar, Elzbieta Korczowska, Janusz Romanek, Roman Zaluska, Janusz Jankielewicz, Dariusz Andrzejewski, Dorota Kruczyk, Przemyslaw Chojnowski, Wojciech Rogowski, Adam Komlo, Jaroslaw Hiczkiewicz, Wojciech Faron, Lucyna Swiatkowska-Byczynska, Wojciech Wieczorek, Lech Kucharski, Joanna Araminowicz, Diana Kociolek, Agnieszka Orda, Adriana Jaremczuk-Kaczmarczyk, Marcin Gruchala, Bartosz Curyllo, Anna Frankiewicz, Piotr Minc, Justyna Rychta, Jolanta Niedek, Halina Krystyna Kowalczyk, Karol Cieslak, Maciej Olszewski, Pawel Szalecki, Grzegorz Kania, Pawel Wojewoda, Agnieszka Sidor, Malgorzata Krzciuk, Piotr Walasik, Anna Szpotowicz, Anna Barszcz, Malgorzata Ozgowicz, Zbigniew Lajkowski, Bozenna Ostrowska-Pomian, Agata Krzesiak-Lodyga, Ewelina Kowal, Anna Jackun-Podlesna, Katarzyna Kalin, Jerzy Lewczuk, Marzena Stopyra-Poczatek, Agata Kaczmarzyk-Radka, Magdalena Wilgat-Szecowka, Renata Romaszkiewicz, Daniel Blaszczyk, Elzbieta Zinka, Agata Pawelska-Buczen, Karolina Zakutynska-Kowalczyk, Anita Biernacka, Agnieszka Karczmarczyk, Robert Kaliszczak, Malgorzata Chmielnicka-Pruszczynska, Dorota Lesniewska-Krynska, Iwona Wozniak-Skowerska, Maria Trusz-Gluza, Seweryn Nowak, Grzegorz Opolski, Agnieszka Kolodzinska, Marek Kiliszek, Marek Bronisz, Jacek Szafranski, Artur Jarzebowski, Malgorzata Brzustowska, Marcin Mielcarek, Marcin Ogorek, Nikolina Roszczyk, Dorota Rozewska-Furmanek, Pawel Ptaszynski, Grazyna Glanowska, Przemyslaw Wilczewski, Piotr Ruszkowski, Jerzy Leszczynski, Monika Smichura, Mateusz Splawski, Grzegorz Skonieczny, Krystyna Jaworska, Lukasz Bernat, Anna Raczynska, Malgorzata Troszczynska, Agnieszka Metzgier-Gumiela, Maria Jaworska-Drozdowska, Tomasz Traczyk, Grzegorz Trzcinski, Ryszarda Piotrowicz, Ryszard Sciborski, Ewa Bekieszczuk, Beata Dolecka, Joanna Starak-Marciniak, Boguslaw Okopien, Malgorzata Klata, Iwona Kobielusz-Gembala, Marcin Basiak, Witold Szkrobka, Tomasz Dybala, Piotr Kukla, Wojciech Kurdzielewicz, Elzbieta Broton, Maciej Kluczewski, Marcin Czamara, Krzysztof Galbas, Agata Markiewicz, Krzysztof Cymerman, Alina Cieszynska, Jaroslaw Jurowiecki, Monika Machnikowska, Jolanta Neubauer-Geryk, Sebastian Tybura, Monika Figura-Chmielewska, Pawel Miekus, Waldemar Myszka, Agata Nowak, Aleksandra Wierzbicka, Karolina Majewska, Stanislaw Mazur, Ewa Miedlar, Monika Ambicka, Karolina Bugajska, Anna Starok, Artur Chmielowski, Michal Mazur, Roman Lysek, Grazyna Jaguszewska, Aleksandra Lysek-Jozefowicz, Grazyna Szumczyk-Muszytowska, Lukasz Wojnowski, Jacek Baszak, Bogdan Walko, Malgorzata Jargiello-Baszak, Wojciech Brzozowski, Teresa Rusicka-Piekarz, Andrzej Galaj, Malgorzata Zyczynska-Szmon, Waldemar Gadzinski, Joanna Luka, Rafal Cichomski, Marian Krzyzanowski, Grzegorz Raczak, Ewa Staniszewska, Pawel Gutknecht, Agnieszka Niemirycz-Makurat, Michal Wrobel, Ewa Kochanska, Mateusz Sciborski, Sebastian Tybura, Ewa Lewicka, Ludmila Danilowicz-Szymanowicz, Malgorzata Szwoch, Monika Figura-Chmielewska, Jolanta Skalska, Barbara Opielowska-Nowak, Lukasz Drelich, Justyna Kabat, Beata Dudzik-Richter, Ewa Domanska, Malgorzata Guziewicz, Dorota Kustrzycka-Kratochwil, Malgorzata Sukiennik-Kujawa, Jadwiga Nessler, Ewelina Lichota, Ewa Gasior, Marta Wegrzynowska, Pawel Rostoff, Rafal Mariankowski, Jozef Lesnik.

Russia

Roman Libis, Irina Kulchenkova, Natalia Shkatova, Gadel Kamalov, Dmitry Belenky, Olga Volodicheva, Olga Kungurtseva, Liudmila Egorova, Alexander Khokhlov, Olga Sinitsina, Svetlana Speshilova, Svetlana Chugunnaya, Elena Lileeva, Eduard Yakupov, Dmitry Zateyshchikov, Irina Zotova, Olga Barbarash, Evgeniya Zhuravleva, Elena

Gorbunova, Tatyana Gorshkova, Yulia Belenkova, Tatiana Kupriyanova, Olga Miller, Olga Kungurtseva, Marina Blinkova, Evgeniy Mazur, Dmitry Platonov, Yurij Orlov, Konstantin Zrazhevskiy, Tatyana Novikova, Fatima Bitakova, Yulia Moiseeva, Elena Polkanova, Konstantin Sobolev, Svetlana Erofeeva, Maria Rossovskaya, Yulia Shapovalova, Ashot Agakhanyan, Alla Kolesnikova, Konstantin Nikolaev, Alla Ovsyannikova, Oksana Zemlianskaia, Elizaveta Panchenko, Ekaterina Kropacheva, Anna Zateyshchikova, Ekaterina Volchkova, Victor Kostenko, Sergey Popov, Roman Batalov, Maria Poltavskaya, Anait Dumikyan, Olga Machilskaya, Anton Edin, Tamara Kolesova, Elena Aleksandrova, Olga Kropova, Oksana Drapkina, Vladimir Ivashkin, Olga Korneeva, Alexander Vishnevsky, Pavel Karchicyan, Oleg Nagibovich, Galina Nagibovich, Galina Monako, Elena Kuznetsova, Petr Chizhov, Elena Novikova, Tatyana Medvedeva, Yulia Ivanova, Marina Gurmach, Svetlana Rachkova, Olga Lebedeva, Elena Shutemova, Svetlana Romanchuk, Margarita Ovchinnikova, Mikhail Sergeev, Daria Konyushenko, Polina Kuchuk, Borys Kurylo, Alexey Ushakov.

Spain

Xavier Vinolas, Enrique Rodriguez F, Jose Mara Guerra, Concepcion Alonso, Marcos Rodriguez Garcia, Douglas Alvarez, Anna Espallargas, Enrique Pena Garcia, Francisco Mendez Zurita, Pere Alvarez Garcia, Nieves Palomo Merchan, Vianeth Rios, Maria Fernanda Lopez Fernandez, Beatriz Herrero Maeso, Maria del Carmen Gomez Castillo, Marta Fernandez, Diego Otero Tomera, Javier Batlle Fonrodona, Joana Costa Pinto Prego de Faria, Teresa Martinez, Luis Tercedor Sanchez, Alejandro Molina Leyva, Rosa Macias, Manuel Molina, Miriam Jimenez, Francisco Jose Bermudez Jimenez, Daniel Castro Fernandez, Maria Jose Jimenez Fernandez, Mercedes Cabrera Ramos, Miguel Alvarez, Maria Molina Jiménez, Carolina Torres, Salvador Tranche Iparraguirre, Norberto Sierra, Joaquin Aracil Villar, Pere Toran Monserrat, Antonio Negrete Palma, Stela Sanchez Parra, Josep Sorribes Lopez, Emilio Marquez Contreras, Jordi Isart Rafecas, Stela Sanchez Parra, Juan Motero Carrasco, Pablo Garcia Pavia, Mateo Cordoba, Ana Briceno Hinojo, Maria del Carmen Gutierrez del Val, Ariadna Gonzales Segovia, Casimiro Gomez Pajuelo, Manuela Pereda Armayor, Miguel Perez Carasa, Carmela Alonso, Luis Miguel Rincon Diaz, Miguel Castillo Orive, Paz Gonzalez, Irene Lasuncion, Luis Fernando Iglesias Alonso, Carolina Santolaya, Angel Grande Ruiz, Jordi Merce Klein, Miriam Garcia Bermudez, Veronica Quintern, Elisabet Serralvo, Jose Ramon Gonzalez Juanatey, Lilian Grigorian, Pilar Mazon, Venesa Garcia Millan, Ana Seoane Blanco, Maria Moure Gonzalez, Gonzalo Baron Esquivias, Marga Gavira Saenz, Mariela Campo Moreno, Ines Monte Collado, Herminia Palacin Piquero, Stela Sanchez Parra, Carles Brotons Cuixart, Esther Fernandez Escobar, Maria Rodriguez Morato, Carmen Rodrigo, Maribel Martines Mena, Ana Domenech Borras, Maria Teixido Fontanillas, Miguel Casanova Gil, Maria Ubeda Pastor, Aileen Austria, Edelmira Barraquer Feu, Xavier Robiro Robiro, Stela Sanchez Parra, Joan Bayo i Llibre, Carme Roca Saumell, Maria Rosa Senan Sanz, Stela Sanchez Parra, Cecilia Corros Vicente, Norberto Sierra, Manuel Vida Gutierrez, Francisco Epelde Gonzalo, Roser Renom, Carlos Alexandre Almeida Fernandez, Sara Saez Jimenez, Encarnacion Martinez Navarro, Nuria del Val Plana, Gemma Tobajas, Marc Roca, Laia Riquelme Sola, Jordi Isart Rafecas, Enrique Escriva Montserrat, Stela Sanchez Parra, Inma Pareja Ibar, Juan Jose Montero Alia, Pilar Montero Alia, Ana Ferrer, Stela Sanchez Parra, Maria Barreda Gonzalez, Norberto Sierra, Maria Angels Moleiro Oliva, Manel Terns Riera, Roger Codinachs Alsina, Marta Dachs, Anna Bartes, Jose Iglesias Sanmartin, Norberto Sierra, Aida Iglesias Garcia, Mercedes Jimenez Gonzalez, Pilar Montero Alia, Stela Sanchez Parra, Juan Jose Montero Alia, Maria del Mar Rodriguez Alvarez, Eva Calvo Martinez, Anna Cortada Cabrera, Monserrat Olle Borque, Rolando Armitano Ochoa, Martin Cebollada del Misterio, Johan Cabeza Ramirez, Victoria Marina Ortega, Carmen Gines Garcia, Antonio Branjovich Tijuan, Eugeni Fernandez Mas, Stela Sanchez Parra, Benardi Jimeno Besa, Rosa Blanca Munoz Munoz, Juan Herreros Melenchon, Tomas Ripoll Vera, Joan Torres Marques, Javier Fosch, Salvador Marcus, Catalina Melia, Yolanda

Gomez Perez, Manuel Jimenez Navarro, Miguel Lopez, Gloria Millan Vazquez, Natalia Andere, Lola Gomez, Hugo Nelson Orellana Figueroa, Victor Becerra Munoz, Alicia Guerrero Molina, Maria Vazquez Caamano, Marcelo Sanmartin Fernandez, Luisa Sanchez Mendez, Maria Fe Arcocha Torres, Izaskun Prieto, Aranza Manzanal Rey, Ane Elorriaga Madariaga, Gonzalo Marcos Gomez, Carolina Ortiz Cortes, Javier Mendoza Vazquez, Pablo Sanchez Calderon, Luis Enrique Lezcano Gort, Carmen Simon Valero, Gema Cancho Corchado, Maria Isabel Cotilla Marco, Andres Iniguez Romo, Maria Belen Lage Bouzamayor, Alicia Martin Vila, Marta Llobet Molina, Carlos Maria Diaz Lopez, Marisol Amaro, Saleta Barbeira, Pablo Juan Salvadores, Diana Montes, Seila Costas, Miguel Angel Prieto Diaz, Eduardo Hevia Rodriguez, Norberto Sierra.

Sweden

Mårten Rosenqvist, Jan Lindén, Kerstin Henriksson, Micael Elmersson, Arnor Egilsson, Karin Floren, Ulf Börjesson, Gunnar Svärd, Susanna Dzeletovic, Bo Liu, Anders Lindh, Rose-Mari Kangert, Lars-Bertil Olsson, Catarina Grässjö, Mikael Gustavsson, Lars Andersson, Lars Benson, Karina Rosenberg, Ann Mannermyr, Claes Bothin, Ali Hajimirsadeghi, Mina Shayesteh, Ruth Lissledal, Jessica Nyström, Björn Martinsson, Birgitta Jansson, Marianne Ericsson, Åke Ohlsson, Lisa Bastani, Kristina Skoglund, Faris Al-Khalili, Pia Löf, Håkan Lindvall, Ann-Britt Ekstrand, Peter Svensson, Camilla Nilsson, Katarina Thörne, Hans Händel, Pyotr Platonov, Anna Osberg, Fredrik Bernstenand, Orjan Stromqvist, Björn Eriksson, Agneta Lindberg, Annica Olofsson, Ingar Timberg, Mari Stjernberg, Milita Crisby, Helena Aaröe, Katarina Risbecker, Daniel Bengtsberg, Sirin Gudmundsson, Annelie Billger, Jan-Erik Karlsson, Dawid Kusiak, Grzegorz Bonkowski, Annika Koch, Jessika Samuelsson, Agneta Andersson, Marita Millborg, Ann-Marie Ohlin, Lennart Malmqvist, Gull-Britt Eriksson, Susanne Hahn, Johan Engdahl, Lisbeth Andersson, Jörgen Thulin, Carl-Johan Lindholm, Aida Hot-Bjelak, Eva Ahbeck, Marie Aman, Steen Jensen, Katarina Mansson, Carl Thorsen, Per Stalby, Johan Lugnegård, Hakan Ahlmark, Anna Pedersen, Annika Lettenström.

Switzerland

Jan Steffel, Ellen Saga, Stephan Winnik, Johann Debrunner, Daniela Amstutz, Alexander Westphalen, Juerg H. Beer, Alexandra Grau, Isberg Henriette, Christian Gustav Lutz, Kirsten Steden, Laura Boos, Philipp Baumgartner, Jana Efe, Marcel Frick, Emanuel Aegerter, Dipen Shah, Jasmine Bruegger, Alexandre Guinand, Guillermet Elise.

Ukraine

Iurii Rudyk, Valeriya Nemtsova, Olena Medentseva, Sergiy Pyvovar, Vira Tseluyko, Olga Romanenko, Oleksandr Karpenko, Liudmyla Todoriuk, Svitlana Kizim, Svitlana Zhurba, Olga Proshak, Igor Kraiz, Ellina Kamenska, Oleksandr Parkhomenko, Oleksandr Shumakov, Iryna Kupnovytska, Irina Kutynska, Roxolana Belegai, Andriy Sapatyi, Nestor Seredyuk, and Roman Petrovskyy, Yuriy Mostovoy, Olha Mostova, Lesia Rasputina, Oleksiy Ushakov, Olena Koval, Pavlo Kaplan, Andrii Ivanov, Igor Kovalskiy, Dmitry Plevak, Yevgeniya Svyshchenko, Larysa Bezrodna, Olena Matova, Tetiana Ovdiienko, Maryna Mospan, Oleg Sychov, Olena Romanova, Mykola Stanislavchuk, Galyna Berko, Liudmyla Burdeina, Oleg Kraydashenko, Vladislav Varenov, Roman Stets, Alina Khmelyova, Andriy Yagensky, Mykhailo Pavelko, Susanna Tykhonova, Olena Khyzhnyak, Ivan Fushtey, Valentyn Mochonyi, Olena Chabanna, Inna Daniuk, Olexandr Palamarchuk.

United Kingdom

Will Murdoch, Naresh Chauhan, Daryl Goodwin, Louise Lumley, Ramila Patel, Philip Saunders, Bennett Wong, Alex Cameron, Philip Saunders, Niranjan Patel, P Jhittay, Andrew Ross, M S Kainth, Karim Ladha, Kevin Douglas, Gill Pickavance, Joanna McDonnell, Laura Handscombe, Trevor Gooding, Helga Wagner, Cumberlidge, Colin Bradshaw, Catherine Bromham, Kevin Jones, Shoeb Suryani, Richard Coates, Bhupinder Sarai, W

Willcock, S Sircar, John Cairns, A Gilliand, Roman Bilas, E Strieder, Peter Hutchinson, Anne Wakeman, Michael Stokes, Graham Kirby, Bhaskhar Vishwanathan, Nigel Bird, Paul Evans, M Clark, John Bisatt, Jennifer Litchfield, E Fisher, Tim Fooks, Richard Kelsall, Neil Paul, Elizabeth Alborough, Michael Aziz, C Ramesh, Pete Wilson, Simon Franklin, Sue Fairhead, Julian Thompson, Hasan Chowan, Gary Taylor, John Wakeling, DawnTragen, Matt Parfitt, Claire Seamark, Carolyn Paul, Mark Richardson, Angus Jefferies, Helen Sharp, Hywel Jones, Claire Giles, Matthew Bramley, Philip Williams, Jehad Aldegather, Simon Wetherell, William Lumb, Phil Evans, Frances Scouller, Neil Macey, Stephen Rogers, Yvette Stipp, Richard West, Philip Pinney, Paul Wadeson, John Matthews, Preeti Pandya, Andrew Gallagher, T Railton, Emyr Davies, Jonathan McClure, Marc Jacobs, Claire Hutton, R Thompson, Bijoy Sinha, Keith Butter, Susan Barrow, Helen Little, David Russell, Ulka Choudhary, Ikram Haq, Paul Ainsworth, Claire Jones, Phil Weeks, Jane Eden, Lisa Gibbons, Janet Glencross, Alison MacLeod, K Poland, Conor Mulolland, A Warke, Paul Conn, D Burns, R Smith, R Kamath, Jonathan Webster, Ian Hodgins, Stephen Vercoe, Paul Roome, Hilary Pinnock, Jayesh Patel, Amar Ali, Nigel Hart, Richard Davies, Nigel De-Sousa, Catherine Neden, Mark Danielsen, Purnima Sharma, Sophia Galloway, Charlotte Hawkins, Raife Oliver, Martin Aylward, Mira Pattni, Gordon Irvine, Shahid Ahmad, Catherine Rothwell, Fiaz Choudhary, Sabrina Khalaque, Stephanie Short, Sharon Peters, Warwick Coulson, Neil Roberts, Amy Butler, Steven Coates, Ben Ward, Daniel Jackson, Steve Walton, Diane Shepherd, Toh Wong, Mark Boon, Melanie Deacon, David Cornelius, Sarah Davies, Ben Frankel, Nick Hargreaves, Henry Choi, Jon Sumner, Tim Myhill, Salah Estifanos, Diane Geatch, Justin Wilkinson, Richard Veale, Karen Forshaw, Rob Hirst, Kashif Zaman, Catherine Liley, Rebecca Wastling Paul McEleny, Andre Beattie, Philip Cooke, Mike Wong, Mark Pugsley, Chaminda Dooldeniya Greg Rogers, James Bennett, Polly Jacobs, Rajesh Muvva, Matthew Adam, Robin Fox, Nicolas Thomas, Simon Cartwright, Rory Reed, Simon Randfield, Christine A'Court, Ann Flynn, Andrew Halpin, Shoeb Suryani Simon Dobson, Louise Lomax, Minnal Nadaph, Iain Munro, Jane Goram, Helen Stoddart, Phil Simmons, John Shewring, Emma Bowen-Simpkins, Mark Rickenbach, Polly Jacobs.

Australia

Adam Blenkhorn, Harry Gibbs, Leverne Hesketh, Janice Boys, Charmaine Morahan, Tracey Mackney, Bhuwanendu Singh, Carol Singh, Monica Campo, Rosemary Beveridge, Laurie McKeon, Elizabeth Vardon, Penny Astridge, Lyne Parsons, Lesley Carlton, Geoffrey Oldfield, William van Gaal, It Meng Tsay, Vicki Lawlor, Chelsea Webster, Damien Cresp, Elizabeth Buckley, Rinku Rayoo, Mary Park, Uwais Mohamed, Naveen Sharma, Vivek Muhta, Elizabeth Vardon, Amie Cho, Walter Abhayaratna, Emily Wilford, Katherine Johnson, Kate Hayes, Natasha Thomas, Renee Eslick, Mehdi Eskandari, Philip Thomson, Vicki OMay, Nathan Dwyer, Catherine McIntosh, Lynette Reid, Teresa Grabek, Karen Patching, Andrew Black, Ben Costello, Rachel Lloyd, George Lukas, Joseph Amin, Ron Lehman, Cameron Singleton, Hazel Morrison, Tanya Patching, Mandy Lehman, Sam Lehman, Jens Kilian, Marika Seremetkoska, Jo-Dee Myers, Deirdre Upton, Alla Waldman, Changjie Song, Joanna Ramachenderan, David Coulshed, Michael Fitzpatrick, Michele MacKenzie, Lisa Barry, George Touma and Inaam Ullah, Andrei Catanchin, Michelle Bonner, Claire Batta, Mary Vorster, Vincent Paul, Nikola Stoyanov, Samantha Thompson, David Colquhoun, Aurelia Connelly, Lara Petelin, Dylan Barnes, Nigel Appleby, Tara Kinnane, Hilary Morrison, Antonio Ferreira-Jardim, Bojana Petrovic, Hosen Kiat, Lisa Wallis, Helena Setio, Anil Aggarwala, Kiran Swaraj, Imran Kassam, David Eccleston and Karen Patching, John French, Maria Plotz, Craig Juergens, Suzanne Raynes, Natalia Sequalino, Kelsey O'Brien, Sally-Anne Hoddy, Tuan Nguyen, Christian Mussap, Hany Dimitri, Krishna Kadappu, Dominic Leung, Alexandra Croucher, Bronte Ayres, Jessie Palmer, Jean Tarrant, Leon Zimmett, Tanya Patching, Marilyn Dolman, Peter Blombery, Helen Rashad, Claire McCarthy, Thanh Phan, Kitty Wong, Lauren Sanders, James Rogers, Bets Conway, Jonathan Sturm, Margaret Webb, Veronica Zenteno, David Crimmins, Anna Schutz,

Susanne Rhodes, David O'Donnell, Karen Patching, Sang Cheol Bae, Harry Gibbs, Vathy Nagalingam, Patrick Carroll, Megan Ratcliffe, Maree Duroux, Samantha Shone, Johnathon Hunter, Mayank H Modi, Richard Geraghty, Greg Starmer, Shane Preston, Michelle Gosley, Sue Richmond, Sue Dixon, Steven Sutcliffe, Margaret Arstall, Katrina Macmillan, Purendra Pati, Jann Parkinson, Jane Rose, Maurits Binnekamp, Jennifer Brimley, Ellie Watson, Prakriti Shrestha, Megan Higgs, Astin Lee, Stephen Mackay, Miyar Prathap Hegde, Taufik Fetahovic, Martin Walker, Joe Famia, Janene Gibbs.

Canada

John Eikelboom, Sam Schulman, Marlene Robinson, Robert Luton, Shannen Douglass, Fraser Gibson, Krystine Cooper, Milan Gupta, Ana Maria Sindilar, Lidia Lepore, Lekraj Nimraj, Mitra Mohammadi, Ranjit Kahlon, Amritanshu Shekhar Pandey, Michelle Pandey, Linda Snell, Stephen Cheung, Jan Kornder, Lynn Breakwell, Tracy Cleveland, Jordana Largy, Alayna Ewert, Shannon Wong, Philippe Beaudry, Kathryn Beaudry Judy Dunnigan, Celine Bergeron, Gordhan Jethoo, Félix Ayala-Paredes, Caroline Lamoureux, Suzanne Maltais, Sonia Nadeau-Lapointe, Veronique Dagenais, Marie-Claude Grenier, Claude Jean, Joseph Berlingieri, Fern Petrie, Stephen Lewis, Chris MacRae, John Heath, Linette Scott, Jane Drown, Germain Poirier, Sandrine Spearson, Emilie Douville, Karine Primeau, Isabelle Neas, Julie Gaudreault, Miranda du Preez, Patricia Roberts, Patti Roberts, Anna Kubanska, Judy Vicic, Nathaly Reynoso, Bradley Schweitzer, Timothy Barkowski, Stephen Burns, Sally Watson, Patti Roberts, Reginald Nadeau, Georgeta Sas, Zahra Djaidani, Sadia Daheb, George Dresser, Donald Farquhar, Richard Kim, Rita Moor, Heather Vosper, Lara Whatmore, Natalie Crown, Ripple Dhillon, Julie Bigcanoe, Nanci Bignell, Tomasz Hruczkowski, Maria Raines, Andrea Lavoie, William Semchuk, Payam Dehghani, Laurie Ferleyko, Cheryl Altwasser, Sheila Kelly, Ratika Parkash, Debbie Wright, Lisa Carroll, Ann Fearon, James Cha, Rebecca Otis, Judy Otis, Elizabeth Burke, Benoit Coutu, Denise Fournier, Julie Fleury, Isabelle Denis, Paul MacDonald, Jeanie MacIsaac, Kathleen Hines, Annette Wood, Christine Hines, Joy Howard, Brian Ramjattan, Wayne Gulliver, Bonnie Clarke, Teri Trahey, Jorge Bonet, Dennis Rupka, Leanne Kwan, Katherine Haveman, Michael Kammermayer, Jacquie Stevenson, Saul Vizel, Beverley Fox, Paul Angaran, Kamran Ahmad, Victoria Korley, Arnold Pinter, Paul Dorian, Iqwal Mangat, Theresa Aves, Sameh Fikry, Wagdy Basily, Ana Maria Jackson.

Egypt

Hany Ragy, Annie Ohanissian, Ahmed Mowafy, Azza Katta, Mazen Tawfik, Moustafa Nawar, Eman Zaatout, Mohamed Sobhy, Ahmed Soliman, Seif Kamal Abou Seif, Tarek Khairy, Ahmed Abd El-Aziz, Michael Sobhy, Nasser Taha, Ashraf Reda, Nevein Sami, Mohamed Reda, Atef Elbahry, Mohamed Setiha, Sameh Samir, Hanan Salem, Mohamed Gamal El Din, Magdi Elkhadem, Ali Mohsen, Adel El-Etreby, Shehab El Etriby, Adham Abdel Tawab.

South Africa

David Kettles, Gerda Du Plessis, Debbie Browne, Penny Jackson, Junaid Bayat, Heidi Siebert, Craig Phillip Franklin, Petronella Nagel, Maria Booysen, Adrian Horak, Elwyn Lloyd, Lindy Henley, Ynez Kelfkens, Riaz Garda, Soraya Cassimjee, Loice Mavhusa, Barry Jacobson, Judy Sasto, Gladys Conway, Leonie Smith, Claire Cannon, Jeannie Marks, Thayabran Pillay, Margo Botha, Michele Guerra, Lelanie van Zyl, Anna-Marie Stapelberg, Deidre Oosthuizen, Louis van Zyl, Marshall Heradien, Judy Anne Finlaison, Tania Ellis, Francois van Zyl, Hendrik Theron, Lorinda de Meyer, Ellen Makotoko, Marinda Karsten, Andonia Page, Andrew Murray, Heather Christie, Cecilia Boshoff, Rikus Louw, Garath Tarr, Annalie Skein, Deon Greyling, Roelof Diederichs, Pindile Mntla, Lillian Rikhotso, Siddique Ismail, Anna Isaacs, Aziza Davids, Mariette Pelser, Sipho Hlengwa, Fayzal Ahmed, Charmaine Chami, Johannes Engelbrecht, Marlene Mostert, Wilhelmina Pretorius, Andrew Ramdass, Muhammed Moosa, Indhranie Naidoo, Shambu Maharajh, Wessel

Oosthuysen, Elizabeth Oosthuysen, Cornel Bester, Mohammed Moosa, Rehana Loghdey, Shireen Safodien, Mishka Salie, Farhaad Shaik, Rosemary Matthews, Veronica Ueckermann, Andre Ueckermann.

United Arab Emirates

Wael Al Mahmeed, Irfan Maqsood, Abdullah Al Naeemi, Maher Makdad, Ghazi Yousef, Ralph Manzano, Karen Magdaluyo, Steve Teves, Nooshin Bazargani, Azbin Abdul, Munther AlOmairi, Amna Al Mulla, Rajan Maruthanayagam, Abdul Rehman, Preetha Haridas, Rupesh Singh, Shereef El Bardisy, Sharmila Jadhav, Preetha Haridas, Ahmed Naguib, Mohamed Ibrahim, Rehab Mohamed, Amrish Agrawal, Jameelah Dominguez, Mukesh Nathani, Ehab M. Esheiba, Nidhi Sharma, Mohammed Thanzeel, Rahul Sharma, Adel Wassef, Rajeev Gupta.

United States

Michael Cox, Erin Malone, Scott Beach, Melanie Eley, April Brown, Cheryl Verdi, Peter Duffy, Tim Richardson, Anne Dickerson, Valerie Palumbo, Shauna Eggertson, Pamela Mason, Stephen Falkowski, Elizabeth Burkett, Tammy Parrott, Karen Johnson, Kevin Ferrick, Ronald Zolty, Sandy Congal, Auris Browne, Miguel Franco, Celine Garcia, Paula Gentry, Melialoha Bartlett, W.Michael Kutayli, Andrew Merliss, Rose Saalfeld, Cheryl Orosco, Nicci Thompson, Annette Quick, Paramdeep Baweja, Daniel Pauly, M. Javed Ashraf, Mickie Keeling, Niraj Sharma, Jowanna Kerr, Marsha Headlee, Vance Wilson, Terry Purcell, Jennifer Langdon, Pamela Jones, Stephen Miller, Scott West, Heather Theobald, Kim Smith, Allison Jones, Lorlie Evans, Mark Alberts, Patricia Knowles, Jan Cameron-Watts, Edwin Blumberg, Susan Paserchia, Roddy Canosa, Elise Hartranft, Susan Felpel, Sarah Jasinski, Ted Gutowski, Susan Karl, Rodney Ison, Tammy Lincoln, Butcher Stephanie, Paula Shaw, Jorge Garcia, Edward Massin, Teresa Hicks, Paul Mullen, Sherry Raziano, Howard Noveck, Fatema Hakimi, Debra Merritt, Abbas Haideri, Pamela Rama, Jill DePauw, Doran Cassidy, Rajneesh Reddy, Melanie Bentley, Marcus Williams, Jin Ah Lee, Deborah Diercks, Daniel Nishijima, Laura Jones, Toni Harbour, Ben Mooso, Allyson Sage, Tirath Sanghera, Michaela Canova, Keith Ferdinand, Patrice Delafontaine, Gholam Ali, Nana Asafu-Adjaye, Suzanne Bowers, Ihsan Haque, Nicole Pickelsimer, Robert Mendelson, Tazrin Tripti, Janaya Raynor, Genna Pearl, Kelly Cervellione, Sridevi Pitta, Kent Davis, Benji Hawkins, Michael Lay, Debbie Fielder, Christopher Herrick, Jay Shoemaker, Daniel Theodoro, Kaharu Sumino, Gregory Ewald, Vanetta Worthy, Anne Thatcher, Lynn Henson, Kelly Ball, Catharine Richard, Jessica Peterson, Charles Treasure, Candy Robertson, Michelle Parker, Lisa Treasure, Joseph Minardo, Moustafa Moustafa, Samuel Emelife, Cas Cader, Sommer Morton, Walter Pharr, Katie Jenkins, Lauren Talton, Bryan Bray, Amy Riddle, Jill Woody, Alisha Oropallo, Farisha Baksh, Sally Kaplan, George Platt, Margaret Thorne, Jeremy Love, Kimberlee Harris, Jaspal Gujral, Peggy Best, Celestine Williams, James Welker, Kathleen Gray, Kristine Wood, Firas Koura, Lori Akers, Margie Duff-Coots.

S1.6. Complete list of ORBIT-AF Principle Investigators

S1.6.1. ORBIT-I Investigators

Nasser Adjei, Alexander Adler, Rahul Aggarwal, Ayim Akyea-Djamson, Anthony Alfieri, Larry Allen, Soufian Al Mahameed, Youssef Al-Saghir, Odilon Alvarado, Salwan Anton, Amy Arouni, Brent Ashcraft, A.A. Aslam, Mahmoud Atieh, Brett Atwater, James Bacon, Eric Batres, Noel Bedwell, Sabrina Benjamin, Peter Berger, John Berry, Ravi Bhagwat, Stephen Bloom, Ronald Blonder, Fernando Boccalandro, Richard Borge, Kevin Browne, Michael Burnam, Randall Burns, Samuel Butman, James Capo, Rene Casanova, Terrance Castor, Tom Christensen, Peter Coats, Kenneth Cohen, Barry Collins, Keith Davis, George Deriso, Robert Detweiler, Andrew Drabick, Matthew Ebinger, Paz Eilat, John

Elsen, Neil Farris, Ramin Farsad, Jeffrey S. Fierstein, Daniel Fisher, Leslie Fleischer, Roy Flood, Augusto Focil, Steven Forman, Donald Fox, Brad Frandsen, Anthony Frey, Mark Gelernt, Santosh Gill, Stanley Golanty, Seth Goldbarg, Charles Gornick, Dina Goytia-Leos, Richard Greengold, Paul Grena, Stephen Grubb, Roger Guthrie, Christopher Hall, Eric Harman, Hoadley Harris, Daniel Hayward, David Hotchkiss, Petar Igic, Henry Ingersoll, John Ip, Timothy Jackson, Glenn Jacobs, Brian Jaffe, Naseem Jaffrani, Ahmad Jingo, Alan Jones, Shaival Kapadia, John Kazmierski, Stephen Kirkland, G. L. Kneller, John Kobayashi, Jay Kozlowski, Jeffrey Kramer, Matthew Krista, Ajay Labroo, Robert Landes, Shuaib A. Latif, Donald Laurion, Charles Leach, Mark Lebenthal, Kenneth LeClerc, Daniel Lee, Michael Lillestol, Ronald Littlefield, William Long, Mark Lurie, Christopher Luttman, Paul Maccaro, George Mallis, Nolan Mayer, Joseph McGarvey, Robert Mendelson, Raman Mitra, J. E. Morriss, John Murray, Ahed Nahhas, Joel Neutel, Abiodun Olatidoye, Abayomi Osunkoya, Benzy Padanilam, Yogesh Paliwal, David Pan, John Pappas, James Poock, Edward Quinlan, John Quinn, Joseph Raffetto, Preet Randhawa, William Reiter, Benjamin Rhee, Eustace Riley, Elvin Rivera-Zayas, Peter Roan, Steven Rosenthal, Steven Rothman, Denis Ruiz-Serrano, Fadi Saba, Matthew Sackett, Ricky Schneider, Michael B. Schwartz, Zachary Seymour, Jeffrey Shanes, Amit Sharma, James Shoemaker, Kenneth Shore, Eugene Silva, Stan Slabic, Victor Simms, Nasser Smiley, David Smith, Calvin Snipes, Brian Snoddy, Harvey Snyder, Rodolfo Sotolongo, Dennis Spiller, Cezar Staniloae, Robert Stein, Steven Stoltz, Stanley Stringam, John Strobel, Damodhar P. Suresh, Gary Sutter, Tahir Tak, Alfred E. Tan, Alan Tannenbaum, Stephen Thew, Troy Thompson, Minang Turakhia, Samir Turk, Kishor Vora, Tomas Vasiliauskas, Andrea Videlefsky, Stephen Voyce, Robert Vranian, Walter Walthall, Randy Watson, Frank Waxman, Debra Weinstein, David Williams, Marcus Williams, Kenneth Wolok, Chakri Yarlagadda, James Zebrack

S1.6.2. ORBIT-II Investigators

Alexander Adler, Gauarv Aggarwala, Syed Ahmed, Joon Ahn, Adeeba Akhtar, M. Akram Khan, Anthony Alfieri, David Allen, Larry Allen, James Allred, Shaik Ali, Odilon Alvarado, Esperanza Arce-Nunez, Mehrdad K Ariani, Brent Ashcraft, Shamaila A. Aslam, Chafik Assal, Mahmoud Atieh, Ruben Ayala, Thomas S. Backer, James Bacon, Mirza Baig, Eric Ball, Subhash Banerjee, Jeffrey Banker, Raed Bargout, Otis Barnum, Tyler Barrett, Aveh Bastani, Chad Bates, Noel Bedwell, Sabrina Benjamin, John Berry, Ravi Bhagwat, Rajesh Bhakt, Juma Bharadia, Jose Birriel, Lawrence Blitz, Stephen Bloom, Fernando Boccalandro, Thomas R. Bowden, Clayton Bredlau, Kevin Browne, Craig Cameron, Roddy Canosa, James Capo, Terrance Castor, Krishnan Challappa, Robert D Chamberlain Jr, Majed Chane, Anna Chang, Rajesh Chawla, Anil Chhabra, John Chin, Chris Christodoulou, Antony Chu, James Cirbus, Christopher Cole, John Covalesky, Ronald D'Agostino, Ram Dandillaya, Kamlesh Dave, Keith Davis, Mark Dawson, George Deriso, Devang Desai, Robert Detweiler, Michael DiGiovanna, Vijay Divakaran, John Dongas, Andrew Drabick, Yamirka Duardo Guerra, C.P. John Earl, William Eaves, Dunbar Matthew Ebinger, Ibrahim Elgabry, Roger Estevez, Victor Farrah, Valerian L. Fernandes, Jason Fleming, Steven Forman, Alex Foxman, James H. French, William J. French, Ira Friedlander, Michael Gabris, Thomas Galski, Raul Garg, Raul Gazmuri, Anil Gehi, Son Giep, Osvaldo Gigliotti, Harinder Gogia, Seth Goldbarg, Jonathan Goldberg, Charles Gornick, Charles Gottlieb, Paul Grena, Harpreet Grewal, John Griffin, Jerrold Grodin, Bernard Grunstra, Roger Guthrie, Ned Gutman, David Harnick, Emil Hayek, John Heath, Christopher Henes, Charles Henrikson, Bengt Herweg, William Herzog, David Hotchkiss, Shawn Howell, Rubina Husain, Mark Huth, Anthony Inzerello, John Ip, Anand Irimpen, Brian Jaffe, Naseem Jaffrani, Preetham Jetty, Nan Jiang, Alan Jones, Rajesh Kabra, Edo Kaluski, Shaival Kapadia, Nikhil Kapoor, Amin Karim, John Kazmierski, Salman Khan, Waqar Khan, James Kmetzo, John Kobayashi, Melissa Kong, Gary Korff, Kannappan Krishnaswamy, Aga Kuliev, Luke Kusmirek, Austin Kutscher, Tak W. Kwan Ajay Labroo, Mark Lebenthal, Daniel Lee, Paul LeLorier, Kurt Lesh, Mark Lieb, Ronald Littlefield,

Jayson Liu, Timothy Logemann, Kevin Longshaw, Roberto Lopez, Mark Lurie, Paul Maccaro, Sharan Mahal, Hector Malave, Amir Malik, Henri Marais, Thor Markwood, Nolan Mayer, Joseph McGarvey, Paul McLaughlin, Robert Craig McPherson, Hitesh Mehta, Viral Mehta, Robert Mendelson, Ivan Mendoza, Fernando Mera, Kevin Merkes, Raman Mitra, Hans Moore, Michael D. Moran, Emily Morawski, Michael T. Morris, J. Edwin Morriss, III, Sandip Mukherjee, Andrew Muller, Basharat Muneer, Allan Murphy, Ramzi Nassif, David Navratil, James D. Nelson, Kent R. Nilsson, John Norris, Howard Noveck, Chibuzo Nwokolo, Benzy Padanilam, Yogesh Paliwal, Jay Pandhi, John Pappas, Herbert Pardell, Leonard Parilak, Hyeun Park, Rakesh Passi, Philip Patel, Steven Pearlman, Severino Pimentel, Craig R. Peterson, James Poock, Kevin Pounds, Thomas Pow, Ahmad Quaddour, Annette Quick, Ofsman Quintana, Jun Anthony Quion, Preet Radhawa, Michael Radin, Joseph Raffett, Luis Ramos, Mayer Rashtian, Chaitany Ravi, Naveed Razzaque, Ram Reddy, Homayoun Reza Ahmadian, Randall Richwine, Lawrence Rink, Elvin Rivera-Zayas, David Rosenbaum, Peter Rouvelas, Steven Rothman, Denis Ruiz Serrano, Walid Saber, Joseph Sacco, Matthew Sackett, Abraham Salacata, Ata T. Salek, Afolabi O. Sangosanya, Howard Sawyer, Aron Schlau, Ricky Schneider, Steven Schwartz, David Scollan, Rakesh Shah, Vipul Shah, Jawad Zar Shaikh, Nicolas Shammas, Jeffrey Shanes, Emanuel Shaoulian, Timothy Shinn, Richard Simons, Dinesh Singal, Narendra Singh, Hicham Siouty, Nasser Smiley, Richard Smith, Calvin Snipes, Sanjiv Sobti, Clark Soderlund, Felix Sogade, Ronald Solbrig, Rodolfo Sotolongo, Shawn Speirs, Dennis Spiller, Jasbir Sra, Bonifasiyo Ssennyamantono, Robert Stein, Albert Sun, Rohit Sundrani, Randeep Suneja, J. Thomas Svinarich, Tahir Tak, Aylmer Tang, Steven Tang, Alan Tenaglia, Raul Torres-Heisecke, Dat Tran, Barry Troyan, Minang Turakhia, Michael Underhill, Tomas Vasiliauskas, Craig Vogel, Vicken Vorperian, Justin Weiner, Ian Weisberg, Stephen Welka, Lindsey White, Jeffrey Whitehill, David Williams, Scott Wilson, Chakri Yarlagadda, Henry Yee, Jerald Young, Jason Zagrodzky

S2. Appendix to Results

Table S1. Interventional procedures included in the analysis

**Procedure** (*N* [%])	**Total**	**Asia**	**Europe**	**Latin America**	**North America**	**Other GARFIELD-AF countries**
Total	7,167 (14.46)	1,540 (10.95)	4,527 (16.18)	302 (7.54)	390 (25.10)	408 (20.71)
Percutaneous coronary intervention	4,234 (59.08)	714 (46.36)	2,819 (62.27)	162 (53.64)	266 (68.21)	273 (66.91)
Coronary artery bypass grafting	300 (16.80)	53 (13.25)	197 (17.80)	13 (18.87)	17 (16.67)	20 (17.65)
Valve replacement	266 (19.00)	36 (29.48)	187 (16.37)	10 (7.95)	14 (18.72)	19 (17.16)
Pacemaker insertion	1,204 (4.19)	204 (3.44)	806 (4.35)	57 (4.30)	65 (4.36)	72 (4.90)
Left atrial appendage closure	76 (3.71)	20 (2.34)	47 (4.13)	2 (3.31)	6 (3.59)	1 (4.66)
Ablation	1,362 (1.06)	454 (1.30)	741 (1.04)	24 (0.66)	73 (1.54)	70 (0.25)
Others	740 (10.33)	207 (13.44)	423 (9.34)	60 (19.87)	26 (6.67)	24 (5.88)
